# Hybrid PEDOT Conductive Polymer-Powdered Metal Sulfide Photocathodes for Photoelectrochemical Green H_2_ Production

**DOI:** 10.1021/acsami.5c15848

**Published:** 2025-12-18

**Authors:** Kengo Nagatsuka, Shunya Yoshino, Yuichi Yamaguchi, Akihiko Kudo

**Affiliations:** † Department of Applied Chemistry, Faculty of Science, 26413 Tokyo University of Science, 1-3 Kagurazaka, Shinjuku-ku, Tokyo 162-8601, Japan; ‡ Carbon Value Research Center, Research Institute for Science and Technology, Tokyo University of Science, Noda-shi, Chiba-ken 278-8510, Japan

**Keywords:** powder-based photocathode, metal sulfide, conductive polymer, green hydrogen, photoelectrochemical cell

## Abstract

Hybrid photocathodes combining metal sulfide powders with poly-3,4-ethylenedioxythiophene (PEDOT) as an effective hole transporting polymer were prepared through a facile drop-casting method and electrochemically oxidative polymerization of 3,4-ethylenedioxythiophene (EDOT). The PEDOT modification remarkably enhanced a photocurrent density of a powder-based (CuGa)_0.5_ZnS_2_ photocathode (bandgap (BG): 2.3 eV) for hydrogen evolution under visible light irradiation at 0 V vs RHE, 60-times higher compared with an unmodified photocathode. The improvement was advantageously effective when using small metal sulfide particles prepared by a flux method compared to large particles synthesized by a solid-state reaction due to improved necking and interfacial contact facilitated by PEDOT in the fine physical structure. The optimized PEDOT/(CuGa)_0.5_ZnS_2_-composited photocathode (PEDOT-(CuGa)_0.5_ZnS_2_) achieved an incident photon-to-current conversion efficiency (IPCE) of 30% at 420 nm and 0 V vs RHE for the H_2_ evolution. PEDOT modification was also effective for Cu_2_ZnSnS_4_ (BG: 1.4 eV) and Cu_3_VS_4_ (BG: 1.5 eV) black metal sulfide photocathodes. Furthermore, solar water splitting proceeded in a two-electrode photoelectrochemical cell consisting of a PEDOT-(CuGa)_0.5_ZnS_2_ photocathode and a BiVO_4_-based photoanode, even without applying an external bias. The photoelectrochemical cell gave 0.08% for nonbias and 0.21% for 0.4 V of an external bias of a solar-to-hydrogen energy conversion efficiency (STH), even though both photoelectrodes were fabricated without any vacuum process and using just two ingredients. Our findings will offer a viable strategy for developing photocathodes that unite simplicity and high performance by using powder materials for the scalable application of photoelectrochemical green H_2_ production.

## Graphical abstract

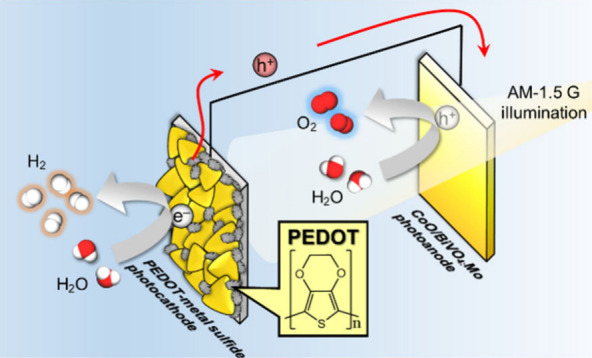

## Introduction

A photoelectrochemical cell for solar water splitting is an attractive device. This system is a promising candidate for green H_2_ productions from H_2_O directly utilizing solar energy for achieving sustainable carbon neutrality.^1–5^ For efficient solar energy conversion, development of visible light-driven photoelectrode is a critical issue. A visible light-driven photoelectrochemical cell can be constructed by combining two types of photoelectrodes. One is a photocathode possessing a p-type semiconducting property such as SrTiO_3_:Rh,^6^ CaFe_2_O_4_,^7^ Cu_2_O,^8^ Cu_3_Nb_1–x_V_x_S_4_,^9,10^ (CuGa_1–y_In_y_)_1–x_Zn_2x_S_2_,^11^ Cu­(In_1–x_Ga_x_)­Se_2_,^12,13^ and Ag_x_Cu_1–x_GaSe_2_ ^14^ for reducing H_2_O to generate H_2_. The other is a photoanode possessing an n-type semiconducting property, for example, (Mo-doped) BiVO_4_,^15–18^ Fe_2_O_3_,^19^ SnNb_2_O_6_,^20^ TaON,^21^ BaTaO_2_N,^22^ Ta_3_N_5_,^23–25^ and CdTe,^26,27^ for oxidizing H_2_O to form O_2_. The photoelectrochemical cell has advantages in terms of an easy separation of gases of reducing and oxidizing productions and the support of an external electronic bias for efficient reactions. Therefore, the development of a highly active photoelectrochemical cell is essential to realizing the practical implementation of artificial photosynthetic systems.

Cu­(I)-containing metal sulfides are excellent materials of photocatalysts that effectively respond to visible light.^1,28,29^ Due to the hybridization of Cu 3d and S 3p orbitals contributing to valence band formation, these materials typically possess a narrow bandgap, a favorable p-type semiconducting property, and a negative conduction band potential. Therefore, numerous studies have reported photoelectrochemical reduction of H_2_O to form H_2_ under visible light irradiation using Cu­(I)-containing metal sulfide photocathodes.^30–34^ Interestingly, some of them can be fabricated by using easily handled photocatalyst powders. One example is a drop-casting method of a simple wet process in which a photocatalyst powder is deposited directly onto a conductive substrate such as a fluorine-doped tin-oxide-coated glass (FTO), an indium tin-oxide-coated glass (ITO), and a carbon paper. However, the powder-based photocathodes often suffer from low photoelectrochemical performance because of interfacial resistances that hinder photogenerated carrier migration between particle-to-particle and particle-to-substrate.^15^ To solve this problem, a particle transfer method reported by Minegishi et al. has often been employed.^35^ This approach enables good electrical contact between photocatalyst particles and the conductive substrate by employing a vacuum evaporation process. For instance, Pt/TiO_2_/CdS/Cu_0.8_Ga_0.4_In_0.4_Zn_0.4_S_2_ ^11^ and Pt/Cu_3_VS_4_ ^10^ photoelectrodes deposited on a Au contacting layer showed an efficient photocathodic performance for H_2_ evolution under simulated sunlight irradiation. Although these photocathodes have an advantage in the high activity of solar H_2_ evolution, the method must utilize a high-vacuum process. Therefore, improving the photocathodic properties of metal sulfide photocathodes without relying on vacuum techniques is significantly challenging in terms of simplicity and cost-effectiveness.

One effective strategy to improve the photocathodic performance is necking between the powder of a photocathode material and a conductive material to suppress interfacial resistance. For example, conductive reduced graphene oxide (RGO) can improve the photoelectrochemical properties of various powder-based photoelectrodes. The conductive material facilitates efficient electron migration and boost charge transport between photocatalyst particles and the conductive substrate electrode, thereby enhancing photoanodic properties of n-type BiVO_4_ and WO_3_ photoanodes and a photocathodic property of a p-type CuGaS_2_ photocathode.^36–38^ In recent years, our research group has also reported the use of conductive organic polymers as modifiers to improve photocathodic properties of powder-based metal sulfide photocathodes.^39,40^ Conductive polymers such as polypyrrole (PPy), poly-3,4-ethylenedioxythiophene (PEDOT), and poly-3,4-ethylenedioxypyrrole (PEDOP) promoted the migration of photogenerated holes from photocatalyst particles to a conductive substrate and drastically improved the photocathodic performance for solar H_2_ evolution in powder-based Cu_1–x_Ag_x_Ga_1–y_In_y_S_2_ photocathodes. Consequently, solar water splitting was successfully achieved utilizing a photoelectrochemical cell consisting of the improved metal sulfide photocathode and a Mo-doped BiVO_4_ (BiVO_4_:Mo) photoanode. Thus, it is strongly expected that further development of powder-based metal sulfide photocathodes modified with conductive polymers, especially PEDOT showing the most effective hole transporting property among the conductive polymers acting as efficient hole transporters, is a promising direction for achieving higher solar H_2_ production efficiency.

We have also reported visible light-driven photocathodes consisting of particulate (CuGa)_0.5_ZnS_2_, Cu_2_ZnSnS_4_, and Cu_3_VS_4_. The (CuGa)_0.5_ZnS_2_ photocathode (bandgap (BG): 2.3 eV) responded to visible light up to 540 nm and was active for solar water splitting.^31,33^ In addition, morphology, photocatalytic performance, and/or photocathodic properties of the metal sulfide powder could readily be tuned by altering the synthetic methods such as a solid-state reaction and a flux method.^33^ On the other hand, Cu_2_ZnSnS_4_ (BG: 1.4 eV)^41^ and Cu_3_VS_4_ (BG: 1.5 eV)^9^ are black metal sulfide photocathodes utilizing the whole range of visible light, offering an advantage for efficient solar energy conversion. There is great interest in enhancing the photocathodic properties of these powder-based metal sulfide photocathodes for H_2_ evolution by incorporating the conductive polymer and material engineering.

In the present study, we developed hybrid photocathodes composed of PEDOT of the conductive polymer serving as an efficient hole transporter and (CuGa)_0.5_ZnS_2_, Cu_2_ZnSnS_4_, and Cu_3_VS_4_ photocatalyst powders for efficient photoelectrochemical H_2_ evolution under visible light irradiation. The mechanisms of improvement in photocathodic performance by PEDOT modification and PEDOT growth on metal sulfide particles were elucidated based on photoelectrochemical performance, morphological characteristics, and electronic band structures. In addition, the versatility of PEDOT modification across various metal sulfide photocathodes was proved. Finally, we demonstrated solar water splitting using a photoelectrochemical cell combining enhanced PEDOT/(CuGa)_0.5_ZnS_2_-composited photocathodes with a CoO-loaded and Mo-doped BiVO_4_ thin-film photoanode.

## Experimental Section

### Preparation of Metal Sulfide Photocatalyst Powders

(CuGa)_0.5_ZnS_2_ (CGZS) photocatalyst powder was prepared by a solid-state reaction (SSR) and a flux method (flux) with LiCl–CsCl mixed salts as previously reports.^31,33^ Cu_2_S (Kojundo Chemical; 99%), Ga_2_S_3_ (Kojundo Chemical; 99.99%), and ZnS (Rare Metallic; 99.99%) as starting materials were mixed in an agate mortar with a molar ratio of Cu:Ga:Zn = 0.5:0.6:1.2. The mixture of starting materials was sealed in a quartz ampule after evacuation and then heat-treated at 1073 K for 10 h to obtain a SSR sample. In other ways, the mixture was sealed in a quartz ampule with a LiCl (Kanto Chemical; 99.0%)–CsCl (Kanto Chemical; 99.8%) (LiCl:CsCl = 3:2, mp: 600 K) flux reagent after evacuation. The sealed ampule was heat-treated at 723 K for 15 h, and then, the obtained sample was washed with pure water to remove the LiCl–CsCl flux reagent. (CuGa)_0.5_ZnS_2_ powder prepared by a solid-state reaction and a flux method are described as (CuGa)_0.5_ZnS_2_ (SSR) and (CuGa)_0.5_ZnS_2_ (flux), respectively.

Cu_2_ZnSnS_4_ and Cu_3_VS_4_ black metal sulfide powders were prepared by a solid-state reaction and a flux method with LiCl–KCl mixed salts following previous reports, respectively.^10,42^ Cu_2_S (Kojundo Chemical; 99%), ZnS (Rare Metallic; 99.99%), SnS_2_ (Kojundo Chemical; 99.99%), and S (Kanto Chemical; 99.5%) as starting materials were mixed in an agate mortar with a molar ratio of Cu:Zn:Sn:S = 1.0:1.15:1.1:0.2 for preparation of Cu_2_ZnSnS_4_. The mixture of starting materials was sealed in a quartz ampule after evacuation and then heat-treated at 973 K for 10 h. In the case of Cu_3_VS_4_ preparation, CuS and V_2_S_3_ starting materials were prepared by ourselves according to a precipitation method by introducing H_2_S gas in an aqueous CuCl_2_ solution (CuCl_2_·2H_2_O, Wako Pure Chemical; 99.0%) and a heat-treating of metallic V (Kojundo Chemical; 99.5%) and S (Kanto Chemical; 99.5%) mixed in a stoichiometric amount at 973 K for 5 h under vacuum, respectively. The mixture of these fresh starting materials with a molar ratio of Cu:V = 3.0:1.1 was sealed with a KCl (Kanto Chemical; 99.5%)–LiCl (KCl:LiCl = 2:3, mp: 625 K) flux reagent, heat-treated at 773 K for 15 h under vacuum, and washed with pure water as mentioned above. A Ru (0.5 wt %) cocatalyst functioning as a H_2_ evolution site was loaded on the black metal sulfide particles by photodeposition from RuCl_3_ (Tanaka Kikinzoku; 38–40% as Ru) in an aqueous 0.5 mol L^–1^ K_2_SO_3_ + 0.1 mol L^–1^ Na_2_S solution before fabrication of a photoelectrode.

### Fabrication of Powder-Based Photocathodes and Modification with PEDOT

The powder-based metal sulfide photocathodes were fabricated by a drop-casting method of a simple wet process. The suspension of photocatalyst powder in ethanol (2 mg mL^–1^) was drop-cast onto FTO substrates (Sigma-Aldrich; ∼7 Ω sq^–1^; 1 × 2 cm^2^) and then dried in air. The obtained photocathodes were washed with ethanol and pure water. 3,4-Ethylenedioxythiophene (EDOT) was polymerized on the photocathodes to form PEDOT modification by an electrochemical oxidation.^40^ An acetonitrile solution of 0.2 mol L^–1^ EDOT and 0.1 mol L^–1^ LiClO_4_ as an electrolyte and oxidant was used as the monomer solution. The powder-based photocathode of a working electrode was dipped in the monomer solution with a bare FTO substrate as the counter electrode and Ag/AgCl as the reference electrode. EDOT was polymerized in the monomer solution according to electrochemical oxidation by applying a constant positive current (1 mA cm^–2^) to form PEDOT onto the photocathode-based working electrode. PEDOT-modified metal sulfide photocathodes are described as PEDOT-(metal sulfide) (e.g., PEDOT-(CuGa)_0.5_ZnS_2_ (flux)). The amounts of modified PEDOT (described as the amounts of EDOT monomer [mC cm^–2^]) were controlled by the charge passed during the electrochemical oxidation. PPy was modified to the (CuGa)_0.5_ZnS_2_ (flux) photocathode according to the same electrochemically oxidative polymerization method on PEDOT modification using a pyrrole monomer (Wako Pure Chemical; 99.0%) instead of EDOT. A composite of (CuGa)_0.5_ZnS_2_ (flux) and RGO (denoted as RGO-CGZS) was prepared by photocatalytic reduction of graphene oxide (GO) over CGZS according to a previous report.^38^ Here, 0.2 g of CGZS powder and GO (NiSiNa Materials; TQ-02-10) (5 wt % to CGZS) were dispersed in a 50 vol % of aqueous CH_3_OH (Kanto Chemical; 99.8%) solution (40 mL). The suspension was irradiated with visible light illumination (λ > 420 nm) for 3 h with N_2_ bubbling. The obtained composite was collected by filtration. A powder-based RGO-CGZS photocathode was fabricated using the drop-casting method with an FTO substrate in the same manner as that mentioned above.

### Fabrication of Mo-Doped BiVO_4_ Photoanode

A Mo-doped BiVO_4_ thin-film photoanode (BiVO_4_:Mo) was fabricated by a facile aqueous solution route.^18^ Bi­(NO_3_)_3_·5H_2_O (Kanto Chemical; 99.9%), NH_4_VO_3_ (Kanto Chemical; 99.0%), and (NH_4_)_6_Mo_7_O_24_ (Kanto Chemical; 99.0%) as starting materials were dissolved in an aqueous 6.5 mol L^–1^ HNO_3_ solution with a molar ratio of Bi:V:Mo = 100:99.5:0.5 [mmol L^–1^] to make a precursor solution. The precursor solution was drop-cast (5 μL cm^–2^) onto an FTO substrate (1 × 2 cm^2^), dried using a heater from the FTO substrate side, and then calcined at 773 K for 2 h in air. A CoO (8 nmol cm^–2^) cocatalyst functioning as an evolution site of O_2_ was loaded on the thin-film photoanode by a drop-casting method. A precursor solution of an aqueous 8.0 × 10^–3^ mol L^–1^ Co­(NO_3_)_2_ (Co­(NO_3_)_2_·6H_2_O, Wako Pure Chemical; 98.0%) solution was deposited onto the photoanode and then calcined at 673 K for 1 h in air.

### Characterization

Crystal phases of the obtained photocathodes were confirmed using X- ray diffraction (XRD) (Rigaku; MiniFlex600, Cu Kα, step size: 0.02°). Diffuse reflectance spectra were obtained using a UV–vis-NIR spectrometer (JASCO; V-780) attached with an integrating sphere and were transferred from reflection to absorbance using the Kubelka–Munk method. The ionization potential corresponding to the valence band maximum and work function was analyzed by photoelectron yield spectroscopy (PYS) (Bunkoukeiki; BIP-KV100, light source: D_2_-lamp) under vacuum. The molecular structure of PEDOT modified on the photocathode was evaluated by laser Raman spectroscopy (JASCO; RMP-5300), employing λ = 785 nm as the excitation source. The top-view and cross-sectional images of the powder-based photocathodes were observed using a field emission-scanning electron microscope (FE-SEM) (JEOL; JSM-6700F). Elemental composition of the photocathode was analyzed using an energy dispersive X-ray spectrometer (SEM-EDS) (JEOL; JED-2300). Surface elemental composition of the photocathode was analyzed by X-ray photoelectron spectroscopy (XPS) (Shimadzu; ESCA-3400, Mg Kα). The binding energies of each spectrum were corrected by reference to the Sn 3d_5/2_ peak (486.5 eV for SnO_2_) detected from an FTO substrate.

### Sacrificial H_2_ Evolution (Half Reaction of Water Splitting)

Photocatalytic H_2_ evolutions in the presence of K_2_SO_3_ (Kanto Chemical; 95.0%) and Na_2_S (Kanto Chemical; 98.0–102.0%) as sacrificial reagents were carried out using a top-irradiation reaction cell attached with a Pyrex window connected to a gas-tight circulation system. The photocatalyst powder (0.3 g) was suspended in an aqueous 0.5 mol L^–1^ K_2_SO_3_ + 0.1 mol L^–1^ Na_2_S solution (150 mL), and the suspension was irradiated with visible light. The irradiated area was approximately 32 cm^2^. A 300 W Xe-arc lamp (PerkinElmer; CERMAX PE300BF) attached with a long-pass filter (HOYA; L42) was used as the light source. The amount of evolved H_2_ was determined using an online gas chromatograph (Shimadzu; GC-8A, MS-5A column; TCD, Ar carrier).

### Photoelectrochemical Measurements

Photocathodic properties for H_2_O reduction to form H_2_ were evaluated utilizing a three-electrode system consisting of a working electrode, a Pt counter electrode, and a Ag/AgCl (TOA-DKK; HS-205C) reference electrode connected with a potentiostat (Meiden Hokuto; HSV-110 or HZ-5000) using a conventional H-type cell separated with a Nafion membrane. An aqueous 0.1 mol L^–1^ K_2_SO_4_ (Kanto Chemical; 99.0%) solution with a phosphate buffer (pH 6.7–8.0) degassed by Ar or N_2_ bubbling was used as an electrolyte. The visible light source was the same Xe-arc lamp (λ > 420 nm) as that mentioned above. Photoelectrodes were irradiated with visible light from the FTO substrate side. In the cyclic voltammetric measurements for drawing *J–V* curves of the present study, the term “onset potential” was defined as the potential at which the cathodic photocurrent density reached 10 μA cm^–2^. The amount of evolved H_2_ was determined using an online gas chromatograph. The Faradaic efficiency (FE) for the H_2_ evolution was calculated using eq 1.

1 $$\mathrm{FE}\;\left[\%\right]=\frac{e^{-}{\mathrm{for H}}_{2}\;\mathrm{evolution}\;\left[\mathrm{mol}\right]}{e^{-}\mathrm{for photocurrent}\;\left[\mathrm{mol}\right]}\times 100$$

Incident photon-to-current conversion efficiency (IPCE) for H_2_ evolution was evaluated using a 300 W Xe-arc lamp (Asahi Spectra; MAX-303) attached with a band-pass filter (Asahi Spectra; λ = 400–600 nm, fwhm = 10 nm) for monochromatic light irradiation. The power of the incident light was measured by using a photodiode head (OPHIRA; PD300-UV head and NOVA display). IPCE was calculated using eq 2.

2 $$\mathrm{IPCE}\;\left[\%\right]=\frac{1240\;\left[\mathrm{eV nm}\right]\times \left|J\right|\;\left[{\mu \mathrm{A cm}}^{-2}\right]}{\lambda \;\left[\mathrm{nm}\right]\times P_{\mathrm{mono}}\;\left[{\mathrm{W m}}^{-2}\right]}$$

Here, *P*_mono_ and λ represent the intensity and wavelength of the incident monochromatic light, respectively. *J* is the photocurrent density.

Photoelectrochemical impedance spectroscopy (PEIS) measurements were performed on the metal sulfide photocathodes using an electrochemical measurement system (Meiden Hokuto; HZ-5000) under the same conditions as for photoelectrochemical measurements. They were measured at 0 V vs RHE ± 20 mV with AC voltage over the frequency range from 0.1 to 10^4^ Hz. The obtained PEIS data were fitted by the Levenberg–Marquardt method.

Solar water splitting was carried out using a one-pot photoelectrochemical cell attached with a Pyrex window consisting of a PEDOT-(CuGa)_0.5_ZnS_2_ (2.5 cm^2^) photocathode and a CoO/BiVO_4_:Mo (1.0 cm^2^) photoanode.^11,31^ The photoelectrochemical cell was connected to an Ar-flow system. An aqueous 0.1 mol L^–1^ K_2_SO_4_ solution with a phosphate buffer (pH 8.0) degassed by Ar bubbling was used as an electrolyte. A solar simulator adjusted with AM-1.5 G (Asahi Spectra; HAL-320, 100 mW cm^–2^) was used as the light source. The amounts of evolved H_2_ and O_2_ were determined using an online gas chromatograph. The solar-to-hydrogen energy conversion efficiency (STH) and the ratio of reacted electrons to holes (e^–^/h^+^) were calculated using eqs 3 and 4, respectively.

3 $$\mathrm{STH}\;\left[\%\right]=\frac{J\;\left[{\mathrm{mA cm}}^{-2}\right]\times \left(1.23-\mathrm{applied bias}\right)\left[V\right]}{P_{\mathrm{sun}}\;\left[{\mathrm{mW cm}}^{-2}\right]}\times 100$$

4 $$e^{-}/h^{+}=\frac{2\times \mathrm{a total amount of evolved}\;H_{2}\;\left[\mathrm{mol}\right]}{4\times \mathrm{a total amount of evolved}\;O_{2}\;\left[\mathrm{mol}\right]}$$

Here, *P*_sun_ and *J* represent the solar energy of 100 mW cm^–2^ and the photocurrent density, respectively. The STH was calculated by subtracting the applied bias from the theoretical electrolysis potential of 1.23 V.

## Results and Discussion

### Photocathodic Properties for H_2_ Evolution under Visible Light Irradiation Using PEDOT-(CuGa)_0.5_ZnS_2_ Photocathodes

A PEDOT-modified (CuGa)_0.5_ZnS_2_-powdered photocathode was characterized by SEM, Raman spectroscopy, and XPS. Figures 1 and 2a and b show the photographs and top-view SEM images of (CuGa)_0.5_ZnS_2_ (flux) photocathodes, respectively. Yellow metal sulfide particles with 100–300 nm of the size of (CuGa)_0.5_ZnS_2_ (flux) were uniformly deposited onto an FTO substrate in the photocathode without PEDOT modification (Figures 1a and 2a). After PEDOT modification by electrochemical oxidation of EDOT, the photocathode slightly darkened (Figure 1b). Raman spectra of the treated photocathode exhibited characteristic bands attributed to PEDOT,^43^ whereas the bare sample showed no such signals (Figure S1), confirming that the blackish modifier was PEDOT formed via electrochemically oxidative polymerization. In addition, PEDOT with an amoeba-like morphology was observed on the surface of the metal sulfide particles, forming robust necking between adjacent particles, as shown in the SEM image (Figure 2b). XPS analysis further verified surface coverage by PEDOT (Figure S2). The Cu 2p signal intensity of the PEDOT-(CuGa)_0.5_ZnS_2_ (flux) photocathode obviously decreased compared to the photocathode without PEDOT modification, indicating that the surface of the (CuGa)_0.5_ZnS_2_ (flux) particles was largely covered with modified PEDOT.^44^ Cross-sectional SEM-EDS mapping of the PEDOT-(CuGa)_0.5_ZnS_2_ (flux) photocathode revealed that the PEDOT (carbon signal) was uniformly modified in the vertical direction for about 5–10 μm of its thickness as shown in Figure 3. Similar amoeba-like PEDOT coatings were also observed in PEDOT-(CuGa)_0.5_ZnS_2_ (SSR) (Figure 2c, d), PEDOT-Ru/Cu_2_ZnSnS_4_ (Figure 2e, f), and PEDOT-Ru/Cu_3_VS_4_ photocathodes (Figure 2g, h). These results suggested that the PEDOT formed via electrochemical oxidation constructed necking structures between photocatalyst particles in various powder-based p-type metal sulfide photocathodes.

**Figure 1:**
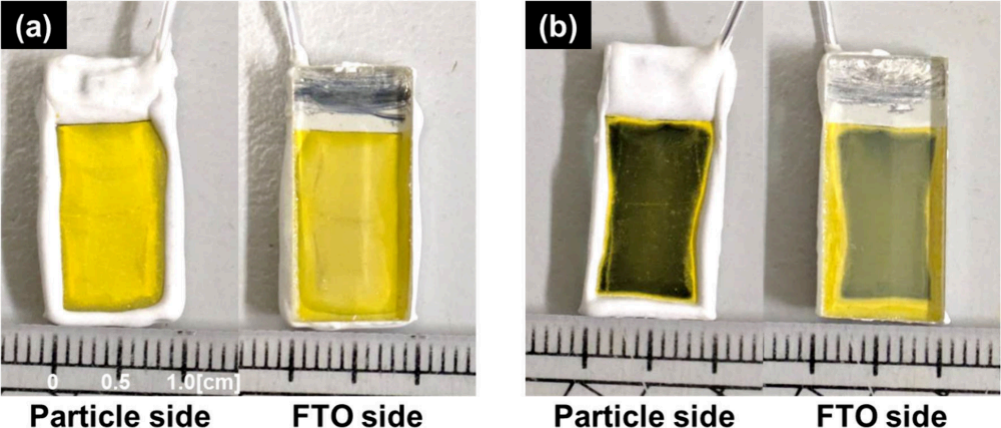
Photographs of (CuGa)_0.5_ZnS_2_ (flux) photocathodes (a) without and (b) with PEDOT modification. Photocatalyst: 0.50 mg cm^–2^; PEDOT: (a) 0, (b) 50 mC cm^–2^.

**Figure 2:**
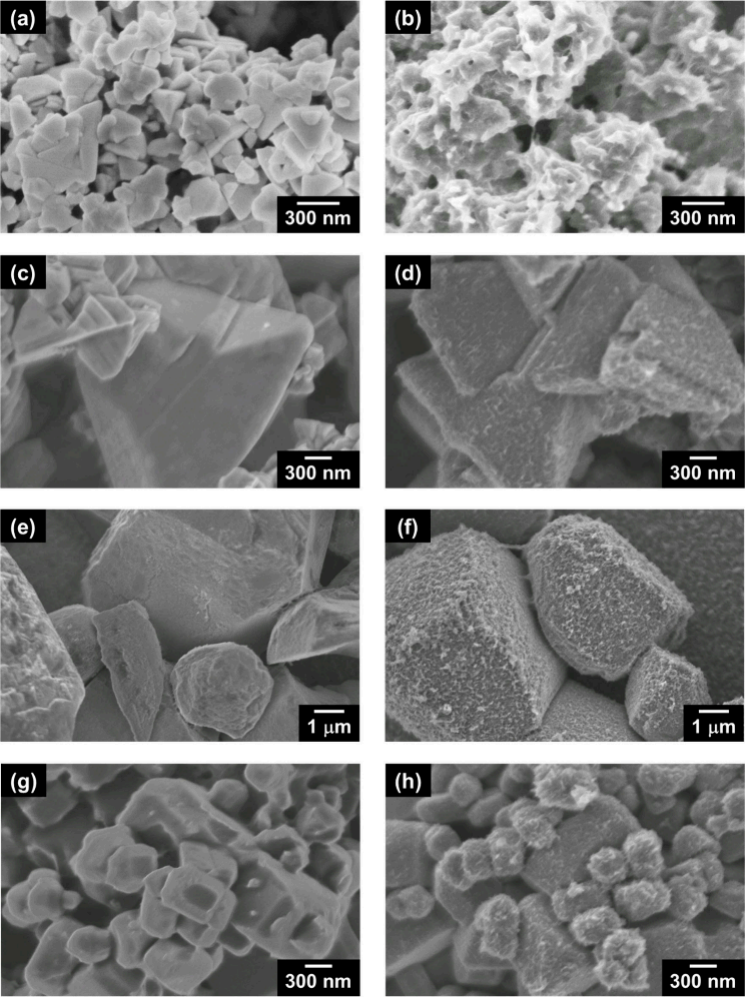
Top-view SEM images of powder-based (a, b) (CuGa)_0.5_ZnS_2_ (flux), (c, d) (CuGa)_0.5_ZnS_2_ (SSR), (e, f) Ru/Cu_2_ZnSnS_4_, and (g, h) Ru/Cu_3_VS_4_ photocathodes. (a, c, e, g) Pristine photocathodes and (b, d, f, h) PEDOT-modified photocathodes. Photocatalyst: (a, b) 0.50 and (c–h) 2.0 mg cm^–2^; PEDOT: (a, c, e, g) 0, (b) 50, and (d, f, h) 40 mC cm^–2^.

**Figure 3:**
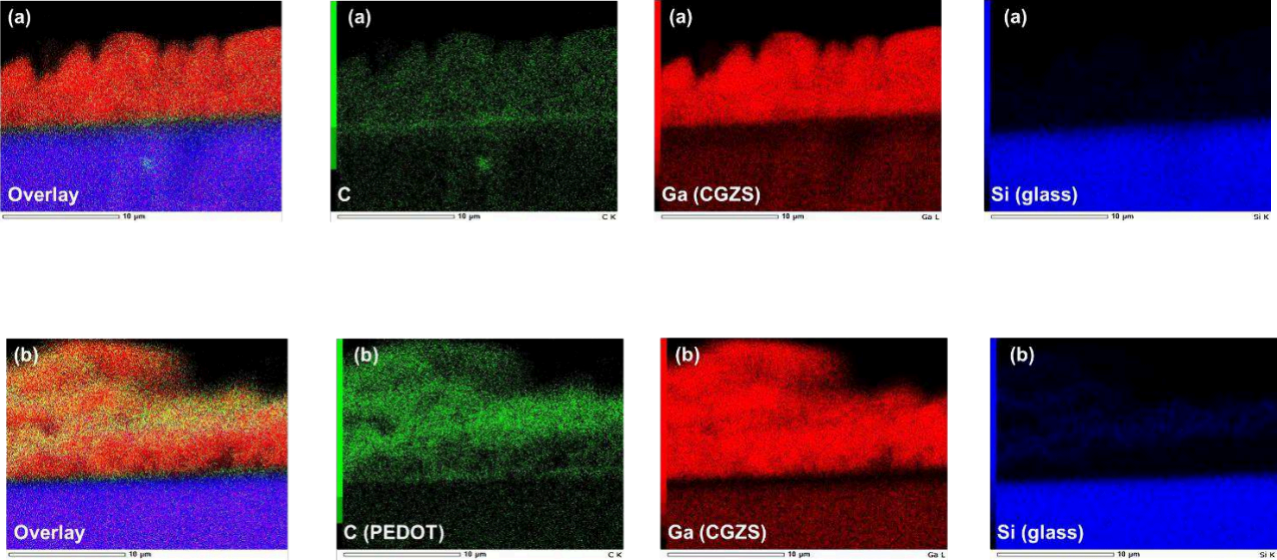
Cross-sectional SEM-EDS mapping images of (CuGa)_0.5_ZnS_2_ (flux) photocathodes (a) without and (b) with PEDOT modification. Photocatalyst: 0.5 mg cm^–2^; PEDOT: (a) 0 and (b) 50 mC cm^–2^.

Figure 4a–b shows *J–V* curves of the (CuGa)_0.5_ZnS_2_ (flux) photocathodes without and with PEDOT modification under visible light irradiation in a N_2_ atmosphere. The photoelectrode without PEDOT modification functioned as a photocathode under visible light, as previously reported^33^ (Figure 4a). Upon PEDOT modification, the cathodic photocurrent density was drastically enhanced more than 60-fold at 0 V vs RHE (Figure 4b). It was notable that H_2_ evolved with approximately 100% of Faradaic efficiency during the photoelectrochemical reaction under an Ar atmosphere as shown in Figure 4c. Modifications with PPy (Figure 4d) and RGO (Figure 4e) were also investigated in comparison with modification effects with PEDOT. The durability for H_2_ evolution under visible light irradiation for 2.5 h of PPy- and RGO-modified (CuGa)_0.5_ZnS_2_ (flux) photocathodes was similar to that of the PEDOT-modified one. In addition, all of the photocathodes showed approximately 100% Faradaic efficiencies for the H_2_ evolution. However, the photocurrent densities of PPy- and RGO-modified photocathodes were only half and 20% of that of the PEDOT-modified photocathode, respectively, indicating that the PEDOT modification was the best in the present study. The photocurrent density more than 2.0 mA cm^–2^ was obtained during a long-term measurement with keeping the FEs of approximately 100% for H_2_ evolution as shown in Figure 5a. Although the photocurrent density gradually decreased, this was reversibly recovered by applying positive bias at +1.0 V vs RHE. However, immediately after this recovery, the subsequent chronoamperometry (CA) measurement exhibited a pronounced photocurrent drop during the first ∼30 min. These behaviors could be explained based on both reversible and irreversible degradations of the photocathode. The reversible degradation was due to an increase in the resistance by reduced PEDOT.^44^ Figure 5b shows diffuse reflectance spectra of the photocathode before and after reaction. Prior to the reaction, the spectrum showed visible light absorption up to 540 nm originating from the (CuGa)_0.5_ZnS_2_ photocatalyst and additional absorption in the visible to near-infrared region attributed to the bipolaronic PEDOT which was an oxidized form.^43,45,46^ After applying the reduction potential at 0 V vs RHE during the reaction, the photocathode gave an additional absorption peak between 550–700 nm which was a characteristic peak of the solitonic PEDOT^43,45,46^ because the polymer was reduced by the negative potential to form a neutral state. When +1.0 V vs RHE potential was reapplied for 10 min under a dark condition, this solitonic absorption disappeared, and the bipolaronic state was reinstated, indicating the recovery of the hole transporting property of the PEDOT. The irreversible degradation would be due to oxidation of Cu­(I) to Cu­(II) in the metal sulfide by anodic polarization in the dark and by photogenerated holes. Since the metal sulfide particles in the photocathode were coated with PEDOT, a chemical state analysis by XPS was not feasible. However, a previous study has shown that prolonged photocatalytic reactions using (CuGa)_0.5_ZnS_2_ led to partial oxidation of the metal sulfide material, resulting in the formation of Cu­(II) species.^47^ The formation of Cu­(II) caused a rapid decrease in the cathodic photocurrent. In contrast, XRD patterns and SEM images of the photocathode before and after the reaction revealed that the crystal structure of the metal sulfide powder and its morphology of the photocathode hardly changed (Figure S3a and b). To probe charge transfer behavior, PEIS measurements were carried out, as shown in Figure 6. Nyquist plots were interpreted using a simplified equivalent circuit model known as the Matryoshka configuration, where high-frequency responses (*R*_1_ *–C*_1_) represented the charge recombination process in the bulk of the photocathode, and low-frequency responses (*R*_2_ *–C*_2_) reflected interfacial charge transfer at the semiconductor–electrolyte junction.^48,49^ The PEIS results suggested a reduction of interfacial resistance between the components of the photocathode such as (CuGa)_0.5_ZnS_2_ particles and (CuGa)_0.5_ZnS_2_–FTO because the PEDOT modification drastically decreased the impedances of the metal sulfide photocathode. Such a remarkable improvement could be attributed to changes in both the physical and band structures, as schematically illustrated in Figure 7. In the case of the unmodified (CuGa)_0.5_ZnS_2_ photocathode, H_2_ evolution under visible light proceeded on the surface of the metal sulfide particles utilizing photogenerated electrons, while photogenerated holes migrated through an FTO substrate to a Pt counter electrode for water oxidation to form O_2_. However, due to significant interfacial resistance between particle-to-particle and particle-to-substrate, most photogenerated holes failed to reach the substrate and the counter electrode, resulting in poor photocathodic performance. In contrast, the PEDOT-(CuGa)_0.5_ZnS_2_ photocathode exhibited efficient hole transport from the metal sulfide particles to the FTO substrate through PEDOT, which significantly reduced interfacial resistance owing to the necked conductive polymer structure^40,44^ (Figure 7a). Furthermore, photoelectron yield spectroscopy was performed (Figure S4)^44^ and revealed that the work function of PEDOT was more negative than the valence band maximum of (CuGa)_0.5_ZnS_2_, forming a stacked band structure between the FTO substrate and the (CuGa)_0.5_ZnS_2_ photocathode for hole transport (Figure 7b). Therefore, PEDOT modification served dual roles as a conductive material and a hole transporter, resulting in a pronounced enhancement of the photocathodic properties for H_2_ evolution under visible light irradiation.

**Figure 4:**
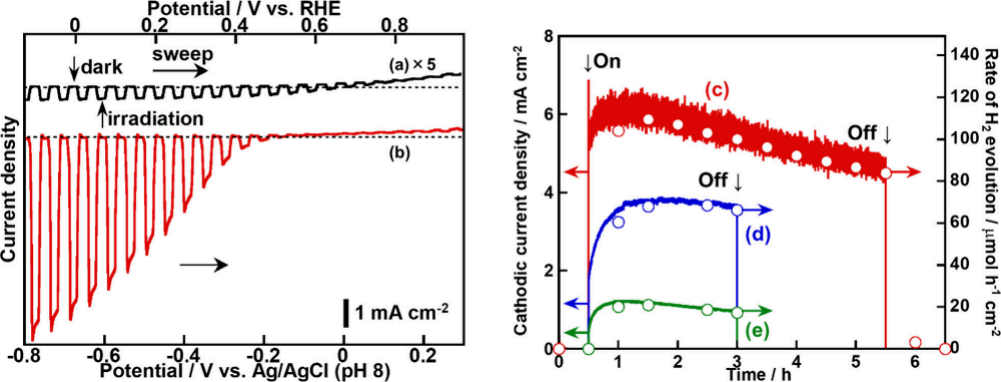
*J–V* curves of (CuGa)_0.5_ZnS_2_ (flux) photocathodes (a) without and (b) with PEDOT modification under visible light irradiation. Photoelectrochemical H_2_ evolution under visible light irradiation using (c) PEDOT-, (d) PPy-, and (e) RGO-modified (CuGa)_0.5_ZnS_2_ (flux) photocathodes. Photocatalyst: 0.5 mg cm^–2^; PEDOT and PPy: 50 mC cm^–2^; RGO: 5 wt %; electrolyte: 0.1 mol L^–1^ K_2_SO_4 (aq.)_ containing a phosphate buffer (pH 7–8) under 1 atm of (a, b) N_2_ and (c–e) Ar gas; CE: Pt wire; RE: Ag/AgCl; (a, b) scan rate: 20 mV s^–1^; (c–e) applied bias: 0 V vs RHE; light source: 300 W Xe lamp (λ > 420 nm), irradiated from an FTO side; cell: H-type cell separated with a Nafion. (c–e) Rate of H_2_ evolution on the right vertical axis describes the theoretical rate corresponding to a cathodic current density on the left vertical axis.

**Figure 5:**
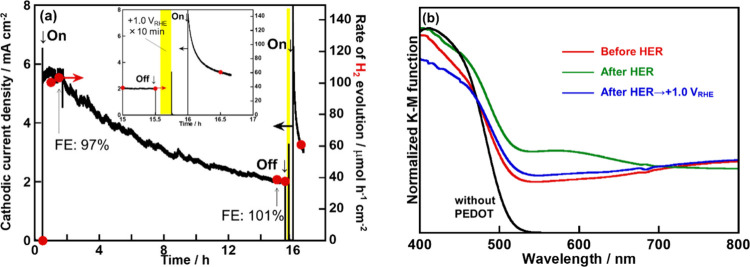
(a) A long-term CA measurement for H_2_ evolution under visible light irradiation using the PEDOT-(CuGa)_0.5_ZnS_2_ photocathode. Photocatalyst: 0.5 mg cm^–2^; PEDOT: 50 mC cm^–2^; electrolyte: 0.1 mol L^–1^ K_2_SO_4 (aq.)_ containing a phosphate buffer (pH 7) under 1 atm of Ar gas; CE: Pt wire; RE: Ag/AgCl; applied bias: 0 V vs RHE; light source: 300 W Xe lamp (λ > 420 nm), irradiated from an FTO side; cell: H-type cell separated with a Nafion. (b) Diffuse reflectance spectra of a PEDOT-(CuGa)_0.5_ZnS_2_ photocathode before and after H_2_ evolution reaction (HER).

**Figure 6:**
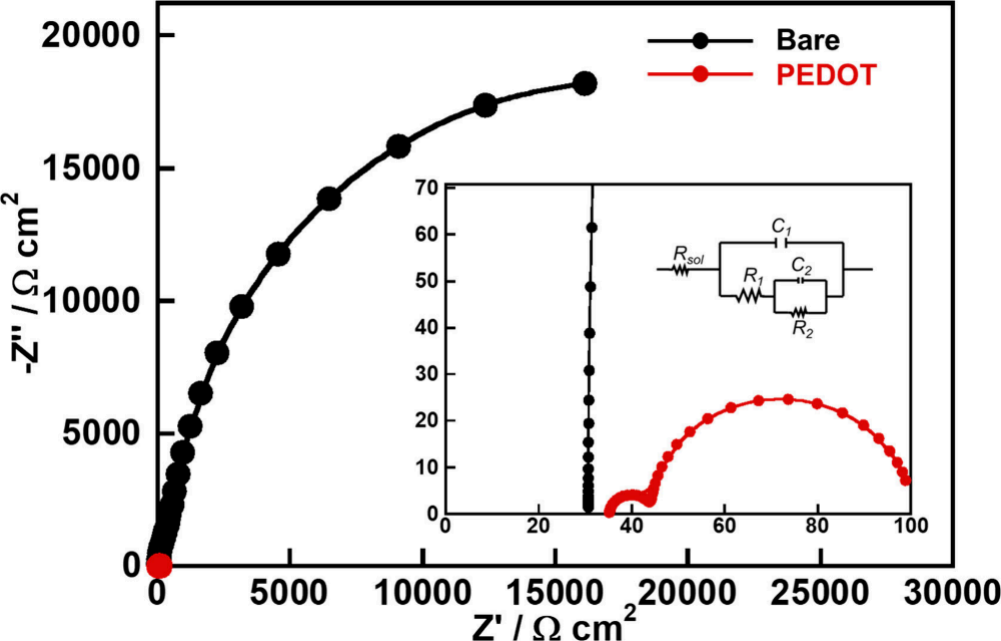
Nyquist plots of (CuGa)_0.5_ZnS_2_ (flux) photocathodes modified with and without PEDOT. Photocatalyst: 0.5 mg cm^–2^; PEDOT: 50 mC cm^–2^; electrolyte: 0.1 mol L^–1^ K_2_SO_4 (aq.)_ containing a phosphate buffer (pH 8.0) under 1 atm of N_2_; CE: Pt wire; RE: Ag/AgCl; applied bias: 0 V vs RHE; amplitude: 20 mV; frequency: 0.1–10^4^ Hz; light source: 300 W Xe lamp (λ > 420 nm), irradiated from an FTO side; cell: H-type cell separated with a Nafion.

**Figure 7:**
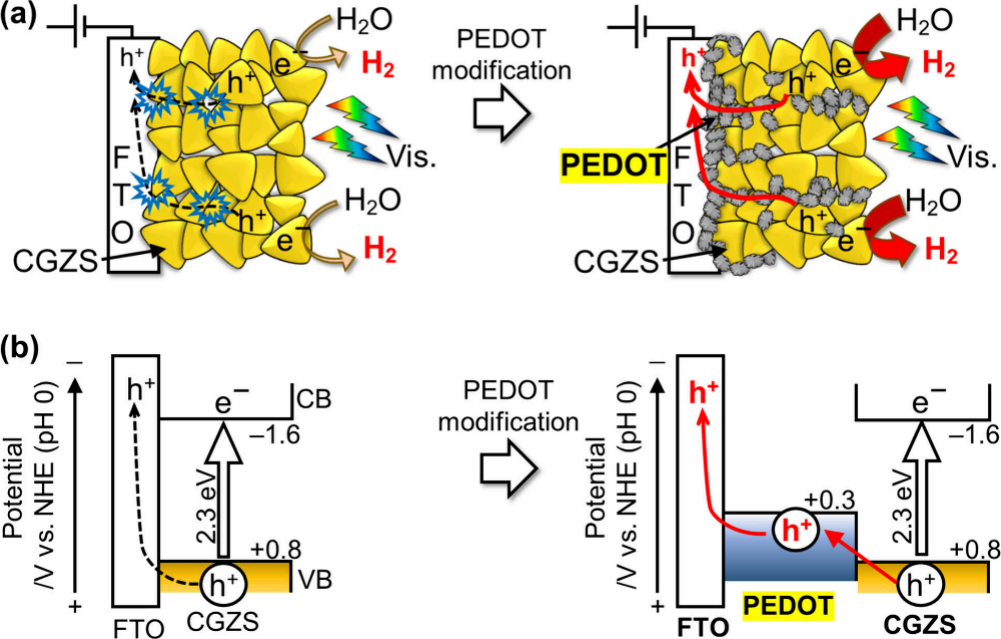
Schematic illustrations of the effect of PEDOT modification on improvement in photocathodic performance of (CuGa)_0.5_ZnS_2_ from the viewpoint of (a) physical and (b) band structure.

Figure 8 shows the effect of an amount of modified PEDOT on the cathodic photocurrent density of a PEDOT-(CuGa)_0.5_ZnS_2_ (flux, 0.50 mg cm^–2^) photocathode. All photocathodes gave higher photocurrent densities under visible light irradiation from an FTO substrate side (black bars in Figure 8) than that from a particle side (white bars in Figure 8). Considering the absorption coefficient of CuGaS_2_ (20,000–50,000 cm^–1^),^50,51^ the light penetration depth is substantially shorter than the thickness of the photocathode films employed in this study (5–10 μm) as shown in Figure 3. This suggested that illuminating the electrode from the FTO substrate side facilitated a more efficient carrier collection, thereby yielding higher photocurrent densities. A volcano-type trend was observed with the highest photocurrent density at 50 mC cm^–2^ of modified PEDOT when the photocathode was irradiated from an FTO side. The amount of PEDOT exerted two competing effects on photocathodic performance: a positive effect by enhancing hole transport and a negative effect by attenuating incident light due to excessive PEDOT coverage, as the conductive polymer was black and coated the photocatalyst surface (Figures 1b and 2b). Thus, the volcano-type trend reflected a trade-off between these two effects. When the photocathode was irradiated from the particle side, the photocurrent increased monotonically with the PEDOT modification amounts. Based on these results, the growth mechanism of the PEDOT modified by electrochemically oxidative polymerization was proposed as shown in Figure 9. Initially, PEDOT grew on the FTO substrate utilizing the anodic current flowing through the conductive layer, filling cavities between the metal sulfide particles and the FTO substrate (Figure 9I). Subsequently, the anodic current flowed through the (CuGa)_0.5_ZnS_2_ particles due to a p-type rectification, leading to direct PEDOT growth on the particle surfaces (Figure 9II). Finally, the PEDOT extended from the bottom to the surface (Figure 9III). By controlling the sequence of anodic current flow, an ideal stacked structure for hole transportation of FTO/PEDOT/(CuGa)_0.5_ZnS_2_ was spontaneously formed as shown in Figure 7b. Although the PEDOT coverage was too dense to be visually discerned, cross-sectional SEM observations confirmed that PEDOT growth in the bottom part of the photocathode became thicker with larger amounts of PEDOT modification (Figure S5). Moreover, direct PEDOT growth on the particle surfaces formed fine necking of the conductive polymer, resulting in enhancement of the photoelectrochemical performance. Actually, a simple-stacked PEDOT/(CuGa)_0.5_ZnS_2_ photocathode prepared by sequential deposition of PEDOT followed by the metal sulfide powder failed to construct such fine necking between the polymer and the particles. Consequently, its photocurrent density was not improved compared with that of the directly grown PEDOT-modified photocathode and even the unmodified photocathode (Figure S6). Thus, the formation of PEDOT/(CuGa)_0.5_ZnS_2_ composites via electrochemical polymerization was the key factor behind the drastic improvements in the photocathodic properties.

**Figure 8:**
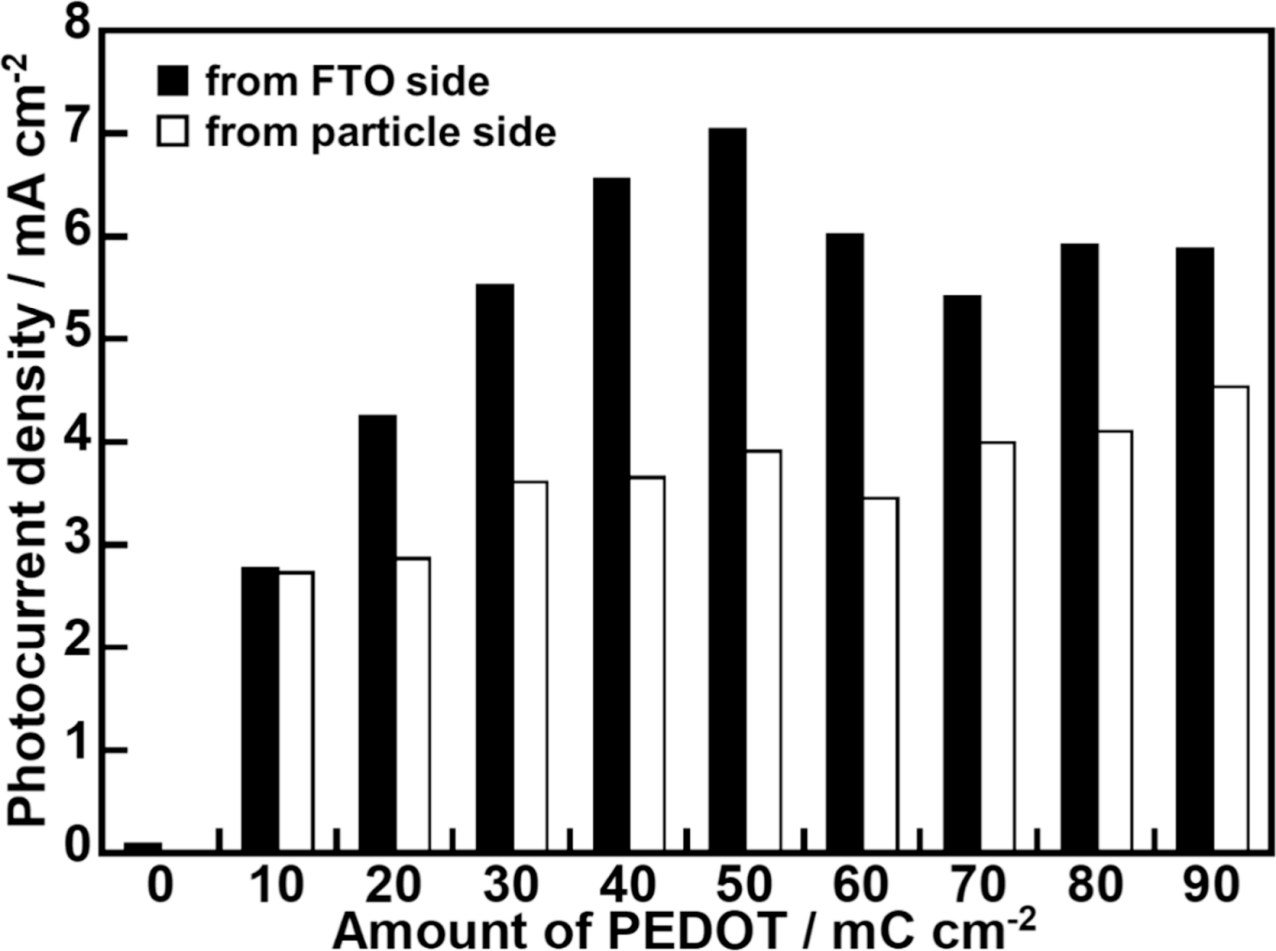
Effect of an amount of modified PEDOT on a cathodic photocurrent density of a PEDOT-(CuGa)_0.5_ZnS_2_ (flux) photocathode under visible light irradiation. Photocatalyst: 0.5 mg cm^–2^; electrolyte: 0.1 mol L^–1^ K_2_SO_4 (aq.)_ containing a phosphate buffer (pH 8.0) under 1 atm of N_2_ gas; CE: Pt wire; RE: Ag/AgCl; applied bias: 0 V vs RHE; light source: 300 W Xe lamp (λ > 420 nm), irradiated from an FTO side; cell: H-type cell separated with a Nafion.

**Figure 9:**
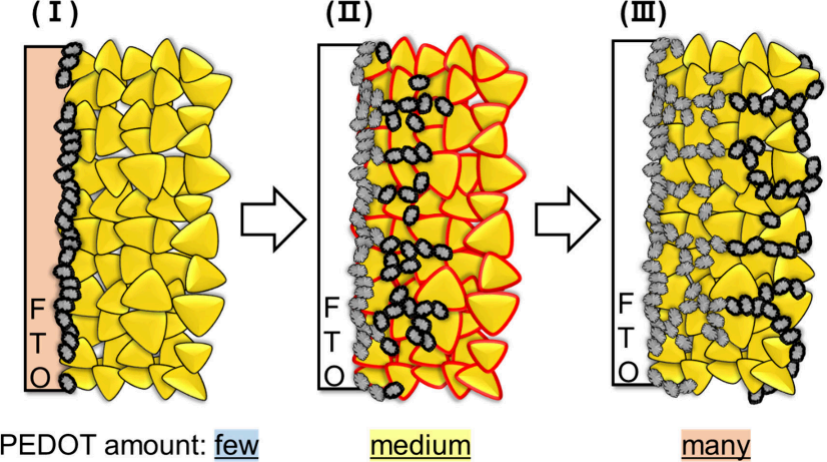
Schematic illustration of how to grow the PEDOT on a powder-based (CuGa)_0.5_ZnS_2_ photocathode by electrochemical oxidation.

Physical structure was a critical factor in determining the photocathodic property of the powder-based photocathode because it affected degrees of necking by PEDOT. Therefore, the effects of the morphology of (CuGa)_0.5_ZnS_2_ particles on the photocathodic property were investigated. Figure 10 shows cross-sectional SEM images of PEDOT-(CuGa)_0.5_ZnS_2_ (SSR) and PEDOT-(CuGa)_0.5_ZnS_2_ (flux) photocathodes with different amounts of deposited photocatalyst powder. Large particles of a SSR sample (1–3 μm) were roughly deposited onto an FTO substrate, resulting in numerous cavities between particle-to-particle and particle-to-substrate (Figure 10a). In particular, when a tiny amount of photocatalyst powder (0.20 mg cm^–2^) was deposited, insufficient particle-to-particle contact was observed due to the scattered large particles (Figure 10b). In contrast, flux-prepared particles composed of the submicrometer particles (100–300 nm) were dispersively deposited (Figure 10c–e), filling the cavities between the particles. This dense deposition was maintained even with significantly reduced powder amounts (0.05–0.20 mg cm^–2^, Figure 10d, e). Table 1 summarizes the photocathodic properties of these photocathodes under visible light irradiation under a N_2_ atmosphere. As mentioned above and previously studied, the metal sulfide photoelectrodes without PEDOT modification exhibited photocathodic activity for H_2_ evolution^31,33^ (entries 1 and 7). The photocurrent densities at 0 V vs RHE significantly increased for both SSR and flux samples by PEDOT modification (entries 2–6, 8–16). The influence of PEDOT modification was further examined in relation to the particle morphology. The highest photocurrent density was achieved at 2.0 mg cm^–2^ of powder deposition for SSR samples (entry 4), whereas it was observed at 0.50 mg cm^–2^ for flux samples (entry 12). A volcano-type trend relying on the amount of photocatalyst powder was due to a trade-off between an increase in photogenerated carriers and surface reaction sites (positive effect) and a decrease in necking by PEDOT due to excessive powder loading and light shielding by excessive thickness (negative effect). Notably, photocathodes with minimal powder loading showed distinct differences between the SSR and flux samples. In SSR, photocurrent density dropped to one-seventh when the powder amount decreased from 2.0 to 0.20 mg cm^–2^ (entries 2–4). In contrast, flux samples maintained photocurrent densities above 5000 μA cm^–2^ across the 0.20–2.0 mg cm^–2^ range (entries 11–14). Even at 0.05 mg cm^–2^ (entry 8), flux samples outperformed SSR samples with 0.20 mg cm^–2^ (entry 2). Additionally, the onset potentials of the flux samples (entries 8–16) were located more positively than those of the SSR samples (entries 2–6). These findings clearly demonstrated that morphology-dependent physical structure was a critical factor in enhancing the performance of powder-based metal sulfide photocathodes via PEDOT modification. In particular, the use of fine-grained powders was advantageous for higher photocathodic performance probably due to constructing a dense PEDOT necking structure.

**Table 1:** Effect of Amounts of Photocatalyst Powders on Photocathodic Properties of PEDOT-(CuGa)_0.5_ZnS_2_ Photocathodes under Visible Light Irradiation^a^

					Cathodic photocurrent density (μA cm^–2^ at 0 V vs RHE)	
Entry	(CuGa)_0.5_ZnS_2_	Amount of photocatalyst (mg cm^–2^)	PEDOT (mC cm^–2^)	Onset potential (V vs RHE)	FTO side	Particle side
1	SSR	2.0	0	0.85	46	4
2	SSR	0.20	40	0.55	610	360
3	SSR	0.50	40	0.58	1750	950
4	SSR	2.0	40	0.55	4010	2290
5	SSR	3.0	40	0.68	2790	1610
6	SSR	4.0	40	0.72	1670	900
7	flux	0.50	0	0.82	102	8
8	flux	0.05	40	0.60	1450	530
9	flux	0.10	40	0.64	2320	1030
10	flux	0.15	40	0.62	4260	2350
11	flux	0.20	40	0.62	6150	3410
12	flux	0.50	40	0.62	6560	3660
13	flux	1.0	40	0.72	5150	2580
14	flux	2.0	40	0.79	5180	1750
15	flux	3.0	40	0.75	1670	190
16	flux	4.0	40	0.75	2460	320

a Electrolyte: 0.1 mol L^–1^ K_2_SO_4 (aq.)_ containing a phosphate buffer (pH 8.0) under 1 atm of N_2_ gas; CE: Pt wire; RE: Ag/AgCl; applied bias: 0 V vs RHE; light source: 300 W Xe lamp (λ > 420 nm); cell: H-type cell separated with a Nafion. Cathodic photocurrent densities were estimated by CV measurements.

**Figure 10:**
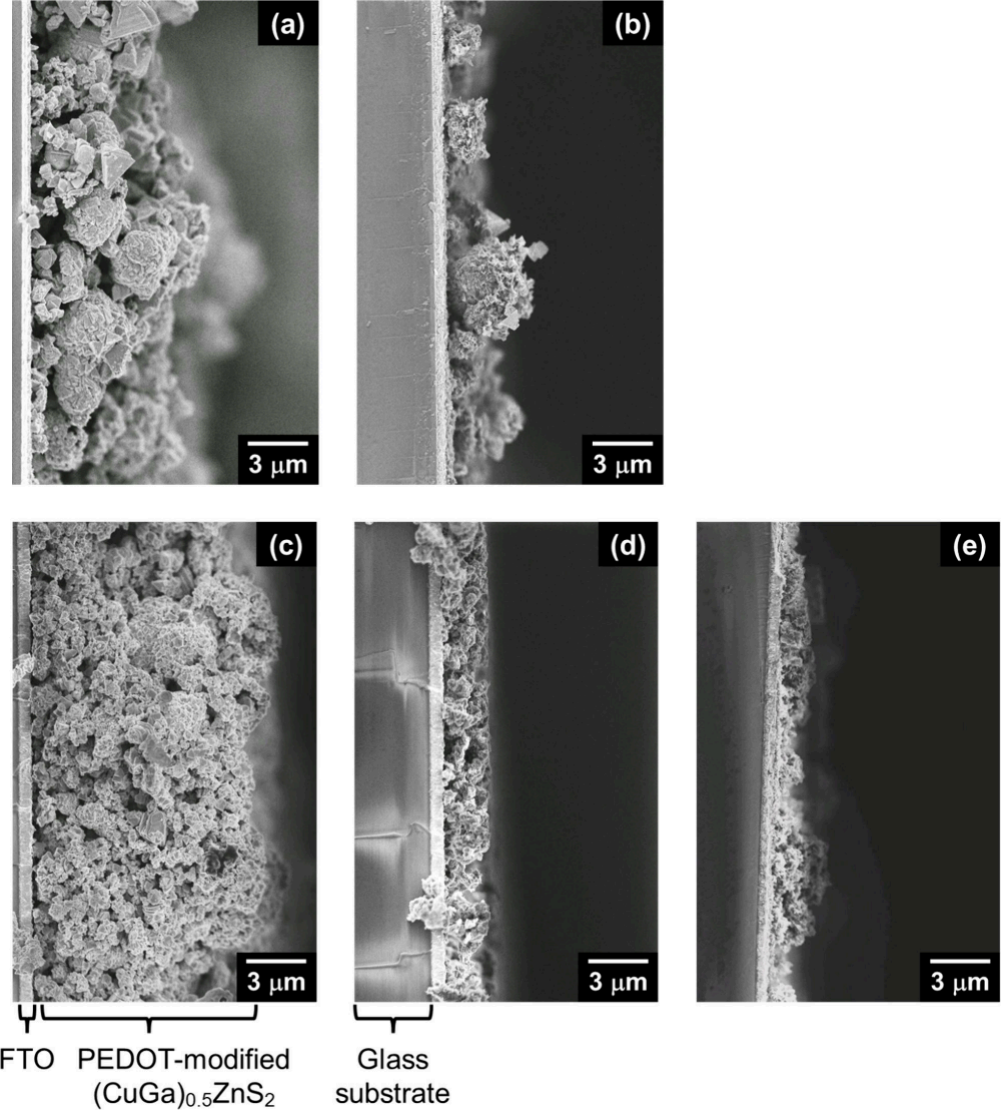
Cross-sectional SEM images of (a, b) PEDOT-(CuGa)_0.5_ZnS_2_ (SSR) and (c–e) PEDOT-(CuGa)_0.5_ZnS_2_ (flux) photocathode. Photocatalyst: (a, c) 2.0, (b, d) 0.20, (e) 0.05 mg cm^–2^; PEDOT: 40 mC cm^–2^.

### Application of PEDOT Modification to Various Metal Sulfide Photocathodes

PEDOT has been demonstrated to function as an effective hole transporter for powder-based (CuGa)_0.5_ZnS_2_ and Cu_1–x_Ag_x_Ga_1–y_In_y_S_2_ photocathodes.^39,40^ To validate the versatility of PEDOT as a hole transporting material further, effects on the photocathodic properties of Cu_2_ZnSnS_4_ ^41^ and Cu_3_VS_4_ ^9^ black metal sulfide photocathodes were also investigated. Table 2 shows photocathodic properties of PEDOT-modified various metal sulfide photocathodes under visible light irradiation in a N_2_ atmosphere. BG was estimated by the absorption edge of the diffuse reflectance spectra (Figure S7). The black metal sulfide photocathodes were loaded with a Ru cocatalyst because they did not show sufficient photoelectrochemical H_2_ evolution activity without cocatalysts.^10^ In contrast, the bare (CuGa)_0.5_ZnS_2_ showed the most effective H_2_ evolution property without any cocatalysts when it was modified with PEDOT as shown in Figure S8 and a previous report.^44^ The photocurrent densities for H_2_ evolution of Ru/Cu_2_ZnSnS_4_ (entries 5 and 6, Figure S9e and f, and Figure S10a) and Ru/Cu_3_VS_4_ (entries 7 and 8, Figure S9g and h, and Figure S10b) photocathodes were also improved by modification with PEDOT as well as (CuGa)_0.5_ZnS_2_ photocathodes (entries 1–4, Figure S9a–d). These results revealed that PEDOT generally worked as a hole transporter across various metal sulfide photocathodes to improve their photoelectrochemical performance. Nevertheless, the enhancement factors by PEDOT modification were modest: only 3.5-fold for Ru/Cu_2_ZnSnS_4_ and 1.2-fold for Ru/Cu_3_VS_4_, compared to the 40–60-fold improvement observed for (CuGa)_0.5_ZnS_2_ photocathodes. It could be presumed that the differences in PEDOT modification effects were caused by the photocatalytic H_2_ evolution activities of the metal sulfide photocatalyst powder. To explore this, sacrificial H_2_ evolution activities under visible light were evaluated in a suspension system as summarized in Table 3. Cu_2_ZnSnS_4_ (entry 3) and Cu_3_VS_4_ (entry 4) exhibited activities approximately 5–10 times lower than those of (CuGa)_0.5_ZnS_2_-type photocatalysts (entries 1 and 2). These results suggested that the lower PEDOT enhancement in black sulfides stemmed from their inherently weaker H_2_ evolution capabilities.

**Table 2:** Photocathodic Properties of PEDOT-Modified Various Metal Sulfides Photocathodes under Visible Light Irradiation^a^

Entry	Photocatalyst	BG (eV)	PEDOT	Cathodic photocurrent density (μA cm^–2^ at 0 V vs RHE)
1	(CuGa)_0.5_ZnS_2_ (SSR)	2.3	×	41.7
2	(CuGa)_0.5_ZnS_2_ (SSR)	2.3	○	1570
3	(CuGa)_0.5_ZnS_2_ (flux)	2.3	×	51.8
4	(CuGa)_0.5_ZnS_2_ (flux)	2.3	○	3080
5	Ru/Cu_2_ZnSnS_4_	1.4	×	6.5
6	Ru/Cu_2_ZnSnS_4_	1.4	○	23.0
7	Ru/Cu_3_VS_4_	1.5	×	84.8
8	Ru/Cu_3_VS_4_	1.5	○	96.7

a Photocatalyst: 2.0 mg cm^–2^; cocatalyst: Ru (0.5 wt %, photodeposition); PEDOT: 40 mC cm^–2^; electrolyte: 0.1 mol L^–1^ K_2_SO_4 (aq.)_ containing a phosphate buffer (pH 8.0) under 1 atm of N_2_ gas; CE: Pt wire; RE: Ag/AgCl; applied bias: 0 V vs RHE; light source: 300 W Xe lamp (λ > 420 nm), irradiated from an FTO side; cell: H-type cell separated with a Nafion. Cathodic photocurrent densities were estimated by CA measurements.

**Table 3:** Sacrificial H_2_ Evolution under Visible Light Irradiation over Various Metal Sulfide Photocatalysts^a^

Entry	H_2_-evolving photocatalyst	BG (eV)	H_2_ evolution (μmol h^–1^)
1	(CuGa)_0.5_ZnS_2_ (SSR)	2.3	1030
2	(CuGa)_0.5_ZnS_2_ (flux)	2.3	1910
3	Cu_2_ZnSnS_4_	1.4	150
4	Cu_3_VS_4_	1.5	400

a Photocatalyst: 0.3 g; cocatalyst: Ru (0.5 wt %, *in situ* PD); solution; 0.5 mol L^–1^ K_2_SO_3_ + 0.1 mol L^–1^ Na_2_S _(aq.)_ (150 mL); light source: 300 W Xe lamp (λ > 420 nm); cell: top-irradiation cell with a Pyrex window; system: gas-tight circulation system.

Figure 11a shows action spectra for the H_2_ evolution of the optimized PEDOT-(CuGa)_0.5_ZnS_2_ (flux) photocathode. The IPCE reached 30% at 0 V vs RHE under irradiation of 420 nm monochromatic light, resulting in approximately three times as high as the previous PEDOT-CuGaS_2_ photocathode (12% at 420 nm).^40^ The IPCE of the present powder-based photocathode was comparable to that of a Pt/TiO_2_/CdS/Cu_0.8_Ga_0.4_In_0.4_Zn_0.4_S_2_/Au photocathode (28% at 450–570 nm) combining with five components fabricated by a particle transfer method based on a vacuum evaporation process.^11^ Thus, a highly active photocathode for H_2_ evolution under visible light irradiation was successfully developed by a facile and nonvacuum deposition technique with only two ingredients during its fabrication process of an electrode. The onset wavelengths of the IPCEs at both 0 and 0.4 V vs RHE agreed well with that of an absorption edge of (CuGa)_0.5_ZnS_2_, indicating the obtained photocurrent originated from the photogenerated carriers of a bandgap excitation of (CuGa)_0.5_ZnS_2_ as shown in Figure 11b.

**Figure 11:**
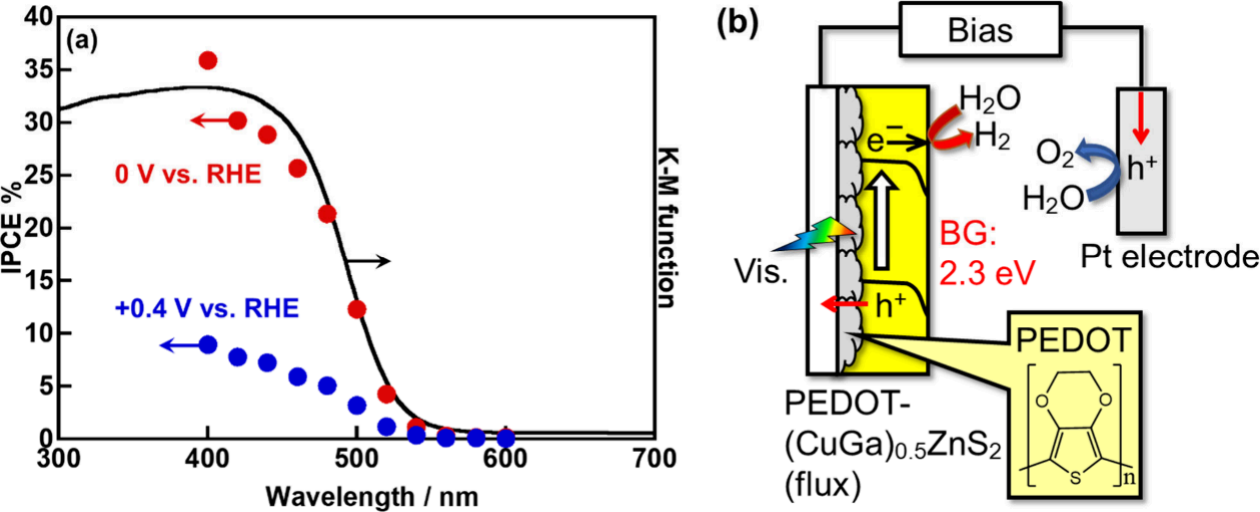
(a) Action spectra of photoelectrochemical H_2_ evolution under visible light irradiation using a PEDOT-(CuGa)_0.5_ZnS_2_ (flux) photocathode. Photocatalyst: 0.5 mg cm^–2^ (0.916 cm^2^); PEDOT: 50 mC cm^–2^; electrolyte: 0.1 mol L^–1^ K_2_SO_4 (aq.)_ containing a phosphate buffer (pH 8.0) under 1 atm of N_2_ gas; CE: Pt wire; RE: Ag/AgCl; applied bias: 0–0.4 V vs RHE; light source: 300 W Xe lamp with band-pass filters (λ = 400–600 nm), irradiated from an FTO side; cell: H-type cell separated with a Nafion. (b) The mechanism of photogenerated carrier migration for photocathodic H_2_O reduction to form H_2_ using a powder-based (CuGa)_0.5_ZnS_2_ photocathode with PEDOT modification.

### Solar Water Splitting Using a Photoelectrochemical Cell Utilizing a PEDOT-(CuGa)_0.5_ZnS_2_ Photocathode

The CoO/BiVO_4_:Mo thin-film photoanode used in this study was the same photoanode as a previously reported paper in our laboratory.^18^ An XRD pattern revealed that a single phase of Scheelite–monoclinic-type BiVO_4_ was successfully obtained on an FTO substrate. Cross-sectional SEM images clarified its thickness of about 600 nm. The photocathode could oxidize H_2_O to form O_2_ under simulated sunlight irradiation at 1.23 V vs RHE, giving nearly 100% of FE. A potential overlap was observed between the cathodic photocurrent of the PEDOT-(CuGa)_0.5_ZnS_2_ photocathodes and the anodic photocurrent of a CoO/BiVO_4_:Mo thin-film photoanode as shown in Figure 12. This overlap suggested that a photoelectrochemical cell combining these electrodes could achieve water splitting without any external bias. The expected photocurrent considered from the crossing point on two *J–V* curves of the photocathode and photoanode under simulated sunlight irradiation was 24 μA cm^–2^ (Figure S11b, c). Photoelectrochemical solar water splitting was performed using a two-electrode cell equipped with an Ar-flow system, as shown in Figure 13A. The sample was irradiated with simulated sunlight from an FTO substrate side of each photoelectrode, sequentially from the photoanode to the photocathode. It was not exactly a tandem device. The extra area of a PEDOT-(CuGa)_0.5_ZnS_2_ photocathode was irradiated with direct light. A part of the PEDOT-(CuGa)_0.5_ZnS_2_ photocathode behind the BiVO_4_ photoanode could absorb the photons transmitted and scattered through the thin-film photoanode that was less than 30% of direct irradiation judging from a transmittance spectrum of a CoO/BiVO_4_:Mo thin-film photoanode (Figure S12). Thanks to the mismatch of the photoelectrode area, nearly 60% of the photocurrent was obtained from the PEDOT-(CuGa)_0.5_ZnS_2_ (flux) photocathode in configuration along a colinear axis with the CoO/BiVO_4_:Mo photoanode (Figure S11c) in comparison with the photocathode without the BiVO_4_-type photoanode (Figure S11a).

**Figure 12:**
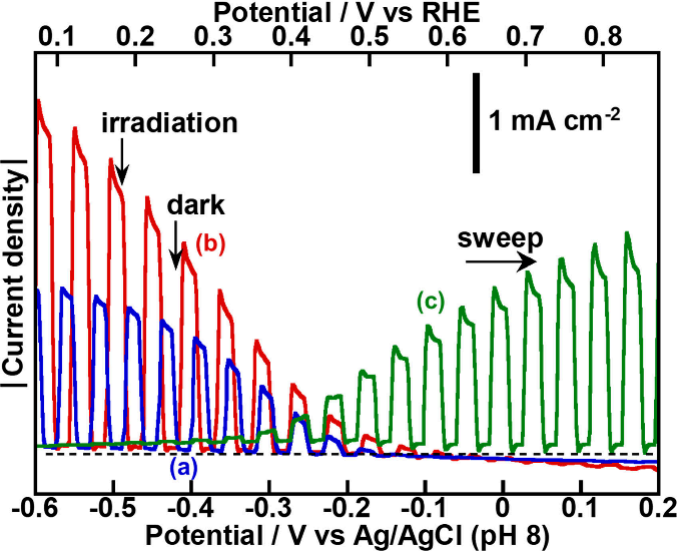
*J–V* curves under visible light irradiation of (a) PEDOT-(CuGa)_0.5_ZnS_2_ (SSR), (b) PEDOT-(CuGa)_0.5_ZnS_2_ (flux) photocathodes, and (c) a CoO/BiVO_4_:Mo­(0.5%) thin film photoanode. Photocathode: 0.5 mg cm^–2^; PEDOT: 50 mC cm^–2^; photoanode: 5.0 μL cm^–2^; cocatalyst: CoO (8 nmol cm^–2^, 673 K-1 h in air); electrolyte: 0.1 mol L^–1^ K_2_SO_4 (aq.)_ containing a phosphate buffer (pH 8.0) under 1 atm of Ar gas; CE: Pt wire; RE: Ag/AgCl; scan rate: 20 mV s^–1^; light source: 300 W Xe lamp (λ > 420 nm), irradiated from an FTO side; cell: H-type cell separated with a Nafion.

**Figure 13:**
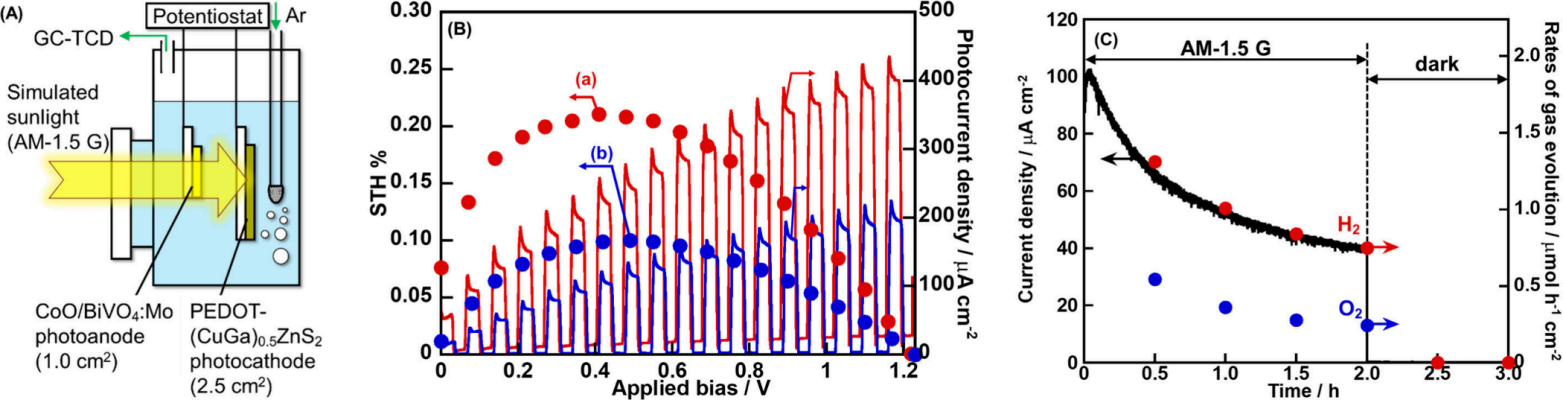
(A) Image diagram of a photoelectrochemical cell for solar water splitting consisting of a PEDOT-(CuGa)_0.5_ZnS_2_ photocathode and a CoO/BiVO_4_:Mo­(0.5%) thin-film photoanode. (B) Solar water splitting using a photoelectrochemical cell consisting of a (a) PEDOT-(CuGa)_0.5_ZnS_2_ (SSR) or (b) PEDOT-(CuGa)_0.5_ZnS_2_ (flux) photocathode and a CoO/BiVO_4_:Mo­(0.5%) thin-film photoanode. (C) Time course of solar water splitting using the photoelectrochemical cell consisting of a PEDOT-(CuGa)_0.5_ZnS_2_ (flux) photocathode and a CoO/BiVO_4_:Mo­(0.5%) photoanode. Photocathode: 0.5 mg cm^–2^; PEDOT: 50 mC cm^–2^; photoanode: 5.0 μL cm^–2^; cocatalyst: CoO (8 nmol cm^–2^, 673 K-1 h in air); electrolyte: 0.1 mol L^–1^ K_2_SO_4 (aq.)_ containing a phosphate buffer (pH 8.0) under 1 atm of Ar gas; (b, c) scan rate: 20 mV s^–1^; (C) applied bias: 0.40 V; light source: solar simulator (AM-1.5 G), irradiated from an FTO side; cell: one-pot cell with a Pyrex window.

The photoelectrochemical cells gave photocurrents for solar water splitting by using both PEDOT-(CuGa)_0.5_ZnS_2_ (flux) (Figure 13B-a) and PEDOT-(CuGa)_0.5_ZnS_2_ (SSR) (Figure 13B-b) photocathodes. The flux sample achieved a maximum STH of 0.21% at an applied bias of 0.41 V, while the SSR sample reached 0.10% at 0.49 V. These values were approximately 10 times higher than the previously reported STH of 0.029% at 0.4 V using a photocathode without PEDOT modification,^31^ confirming the effectiveness of PEDOT modification in solar water splitting. Remarkably, solar water splitting also proceeded even without any external bias. The photocurrent value in the case using the flux sample (52 μA cm^–2^) was not very far from the expected photocurrent, as mentioned above. The STH under zero-bias conditions was 0.08% for the flux sample, which was significantly higher than the 0.01% observed for the SSR sample. These results indicated that the PEDOT-(CuGa)_0.5_ZnS_2_ (flux) photocathode composed of fine particles facilitated more efficient solar water splitting than the SSR counterpart with rough particles. This enhancement was attributed to the more positive onset potential of the flux sample, which provided a broader overlap with the photocurrent of the CoO/BiVO_4_:Mo photoanode (Table 1 and Figure 12). Figure 13C shows a time course of solar water splitting by the photoelectrochemical cell utilizing a PEDOT-(CuGa)_0.5_ZnS_2_ (flux) photocathode. H_2_ and O_2_ evolved, corresponding to the photocurrent under simulated sunlight irradiation at 0.40 V, while no gas evolution or current was detected in the dark. This almost 100% Faradaic efficiency indicated that the photocurrent originated from solar water splitting into H_2_ and O_2_. This result also implied that the obtained photocurrents, as shown in Figure 13B, were also attributable to water splitting reactions. The origin of the instability of the photocurrent was probably due to degradation of the CoO/BiVO_4_:Mo photoanode. Actually, the revived photocurrent was successfully obtained by using a fresh CoO/BiVO_4_:Mo photoanode instead of the degraded old photoanode after solar water splitting.

## Conclusions

The drastic improvement in photocathodic properties of powder-based (CuGa)_0.5_ZnS_2_ photocathodes under visible light irradiation was successfully demonstrated through PEDOT modification via electrochemically oxidative polymerization without requiring any vacuum methods during the modification process. PEIS measurements revealed that the modified PEDOT functioned as a conductive material to reduce interfacial resistance drastically in the bulk of the powdered photocathode. Additionally, DRS and PYS results determined the band structure of the photocathode, clarifying that PEDOT also worked as a hole transporter. The bifunctionality led to facilitating migration of photogenerated holes from the metal sulfide particles to the FTO substrate, resulting in high photocathodic performance for H_2_O reduction to generate H_2_. Fine particles prepared by a flux method proved an advantage over large particles synthesized by a solid-state reaction, as the flux-prepared powder enabled precise incorporation of the conductive polymer and formation of dense necking structures. The optimized PEDOT-(CuGa)_0.5_ZnS_2_ (flux) photocathode exhibited a high IPCE of 30% at 0 V vs RHE under 420 nm monochromatic light irradiation even though the photocathode was fabricated without any vacuum process and using just two ingredients. Furthermore, PEDOT modification also enhanced the photocathodic properties of black metal sulfide photocathodes such as Cu_2_ZnSnS_4_ (BG: 1.4 eV) and Cu_3_VS_4_ (BG: 1.5 eV), demonstrating the versatility of PEDOT as a hole transporting material. Photoelectrochemical solar water splitting was successfully demonstrated using a two-electrode photoelectrochemical cell composed of the hybrid PEDOT-(CuGa)_0.5_ZnS_2_ (flux) photocathode and a CoO/BiVO_4_:Mo photoanode achieving a STH of 0.21%: it was 10 times higher than that of a photocathode without PEDOT. The present reported STH remains modest; however, the water splitting performance is expected to be further enhanced by controlling the physical structure of the powder-based photocathode. The present photocathode was fabricated by a drop-casting method of a facile wet process under ambient conditions. Despite its simplicity, it substantially limits fine control over the morphological powder deposition state including the dispersion of photocatalyst particles, secondary particle formation, surface roughness, and patterning. Further improvement in photocathodic performance is anticipated through refined control of the structure, for instance, constructing gas-diffusion structures to promote the evolution of H_2_ bubbles. The detailed study of controlling its physical structure will be discussed in a separate report. This present study strongly provides a strategy for developing simple and efficient metal sulfide photocathodes without relying on vacuum-based fabrication, paving the way toward scalable artificial photosynthetic systems for green H_2_ production.

## Supplementary Material



## Notes

Raman and XPS spectra of PEDOT-modified (CuGa)_0.5_ZnS_2_ (flux) photocathodes, XRD patterns, and top-view SEM images of PEDOT-(CuGa)_0.5_ZnS_2_ (flux) photocathode before and after a long-term CA measurement; PYS spectra of (CuGa)_0.5_ZnS_2_ (flux) and PEDOT; cross-sectional SEM images of the bottom part of PEDOT-(CuGa)_0.5_ZnS_2_ (flux) photocathodes; photoelectrochemical measurements using a simple-stacked PEDOT/(CuGa)_0.5_ZnS_2_ (flux) photocathode; *J–V* curves of PEDOT-(CuGa)_0.5_ZnS_2_ (flux) photocathode loaded with a Ru cocatalyst under visible light irradiation; cathodic photocurrent densities under visible light irradiation of unmodified and PEDOT-modified various metal sulfide photocathodes; *J–V* curves of PEDOT-modified Ru/Cu_2_ZnSnS_4_ and Ru/Cu_3_VS_4_ blackish photocathodes; diffuse reflectance spectra of various metal sulfide photocatalysts; *J–V* curves under simulated sunlight irradiation of PEDOT-(CuGa)_0.5_ZnS_2_ (flux) photocathodes; CoO/BiVO_4_:Mo­(0.5%) thin-film photoanode and CoO/BiVO_4_:Mo photoanode-stacked PEDOT-(CuGa)_0.5_ZnS_2_ (flux) photocathode; and transmittance spectrum of CoO/BiVO_4_:Mo thin-film photoanode (PDF)

## References

[1] Kudo A., Miseki Y. (2009). Heterogeneous Photocatalyst Materials for Water Splitting. Chem. Soc. Rev..

[2] Osterloh F. E. (2008). Inorganic
Materials as Catalysts for Photochemical Splitting of Water. Chem. Mater..

[3] Setoyama T., Takewaki T., Domen K., Tatsumi T. (2017). The Challenges of Solar
Hydrogen in Chemical Industry: How to Provide, and How to Apply?. Faraday Discuss..

[4] Yamada T., Domen K. (2018). Development of Sunlight Driven Water Splitting Devices towards Future
Artificial Photosynthetic Industry. ChemEngineering.

[5] Kudo A. (2025). Development
of Photocatalysts for Artificial Photosynthesis Aiming at Carbon Neutrality. Electrochemistry.

[6] Iwashina K., Kudo A. (2011). Rh-Doped SrTiO_3_ Photocatalyst Electrode Showing Cathodic
Photocurrent for Water Splitting under Visible-Light Irradiation. J. Am. Chem. Soc..

[7] Ida S., Yamada K., Matsunaga T., Hagiwara H., Matsumoto Y., Ishihara T. (2010). Preparation of p-Type
CaFe_2_O_4_ Photocathodes for Producing Hydrogen
from Water. J. Am. Chem. Soc..

[8] Wick R., Tilley S. D. (2015). Photovoltaic and
Photoelectrochemical Solar Energy
Conversion with Cu_2_O. J. Phys. Chem.
C.

[9] Ikeda S., Aono N., Iwase A., Kobayashi H., Kudo A. (2019). Cu_3_MS_4_ (M = V, Nb, Ta) and Its Solid Solutions
with Sulvanite Structure for Photocatalytic and Photoelectrochemical
H_2_ Evolution under Visible-Light Irradiation. ChemSusChem.

[10] Fukai H., Nagatsuka K., Yamaguchi Y., Iwase A., Kudo A. (2022). Powder-Based
Cu_3_VS_4_ Photocathode Prepared by Particle-Transfer
Method for Water Splitting Using the Whole Range of Visible Light. ECS J. Solid State Sci. Technol..

[11] Hayashi T., Niishiro R., Ishihara H., Yamaguchi M., Jia Q., Iwase A., Minegishi T., Yamada T., Domen K., Kudo A. (2018). Powder-Based
(CuGa_1‑y_In_y_)_1‑x_Zn_2x_S_2_ Solid Solution
Photocathodes with a Largely Positive Onset Potential for Solar Water
Splitting. Sustain. Energy Fuels.

[12] Moriya M., Minegishi T., Kumagai H., Katayama M., Kubota J., Domen K. (2013). Stable Hydrogen
Evolution from CdS-Modified CuGaSe_2_ Photoelectrode
under Visible-Light Irradiation. J. Am. Chem.
Soc..

[13] Kobayashi H., Sato N., Orita M., Kuang Y., Kaneko H., Minegishi T., Yamada T., Domen K. (2018). Development of Highly
Efficient CuIn_0.5_Ga_0.5_Se_2_-Based Photocathode
and Application to Overall Solar Driven Water Splitting. Energy Environ. Sci..

[14] Zhang L., Minegishi T., Kubota J., Domen K. (2014). Hydrogen Evolution
from Water Using Ag_x_Cu_1‑x_GaSe_2_ Photocathodes under Visible Light. Phys. Chem.
Chem. Phys..

[15] Iwase A., Kudo A. (2010). Photoelectrochemical Water Splitting Using Visible-Light-Responsive
BiVO_4_ Fine Particles Prepared in an Aqueous Acetic Acid
Solution. J. Mater. Chem..

[16] Jia Q., Iwashina K., Kudo A. (2012). Facile Fabrication
of an Efficient
BiVO_4_ Thin Film Electrode for Water Splitting under Visible
Light Irradiation. Proc. Natl. Acad. Sci. U.S.A..

[17] Kim T. W., Choi K.-S. (2014). Nanoporous
BiVO_4_ Photoanodes with Dual-Layer
Oxygen Evolution Catalysts for Solar Water Splitting. Science.

[18] Iwase A., Ikeda S., Kudo A. (2017). Efficient Solar Water Oxidation to
Oxygen over Mo-Doped BiVO_4_ Thin Film Photoanode Prepared
by a Facile Aqueous Solution Route. Chem. Lett..

[19] Kim J. Y., Magesh G., Youn D. H., Jang J. W., Kubota J., Domen K., Lee J. S. (2013). Single-Crystalline,
Wormlike Hematite
Photoanodes for Efficient Solar Water Splitting. Sci. Rep..

[20] Niishiro R., Takano Y., Jia Q., Yamaguchi M., Iwase A., Kuang Y., Minegishi T., Yamada T., Domen K., Kudo A. (2017). A CoO_x_-Modified
SnNb_2_O_6_ Photoelectrode for Highly Efficient
Oxygen Evolution from Water. Chem. Commun..

[21] Higashi M., Domen K., Abe R. (2012). Highly Stable
Water Splitting on
Oxynitride TaON Photoanode System under Visible Light Irradiation. J. Am. Chem. Soc..

[22] Ueda K., Minegishi T., Clune J., Hisatomi T., Nishiyama H., Katayama M., Shibata N., Kubota J., Yamada T., Domen K. (2015). Photoelectrochemical Oxidation of Water Using BaTaO_2_N Photoanodes Prepared by Particle Transfer Method. J. Am. Chem. Soc..

[23] Liu G., Ye S., Yan P., Xiong F., Fu P., Wang Z., Chen Z., Shi J., Li C. (2016). Enabling an Integrated
Tantalum Nitride Photoanode to Approach the Theoretical Photocurrent
Limit for Solar Water Splitting. Energy Environ.
Sci..

[24] Zhong M., Hisatomi T., Sasaki Y., Suzuki S., Nakabayashi M., Shibata N., Nishiyama H., Katayama M., Yamada T., Domen K. (2017). Highly Active GaN-Stabilized Ta_3_N_5_ Thin-Film Photoanode for Solar Water Oxidation. Angew. Chem..

[25] Pihosh Y., Nandal V., Higashi T., Shoji H., Yamada T., Nicolosi V., Hisatomi T., Matsuzaki H., Seki K., Domen K. (2023). Tantalum
Nitride-Enabled
Solar Water Splitting with Efficiency Above 10%. Adv. Energy Mater..

[26] Lichterman M. F., Carim A. I., McDowell M. T., Hu S., Gray H. B., Brunschwig B. S., Lewis N. S. (2014). Stabilization of N-Cadmium Telluride
Photoanodes for Water Oxidation to O_2_(g) in Aqueous Alkaline
Electrolytes Using Amorphous TiO_2_ Films Formed by Atomic-Layer
Deposition. Energy Environ. Sci..

[27] Su J., Minegishi T., Kageshima Y., Kobayashi H., Hisatomi T., Higashi T., Katayama M., Domen K. (2017). CdTe-Based
Photoanode for Oxygen Evolution from Water under Simulated Sunlight. J. Phys. Chem. Lett..

[28] Takayama T., Tsuji I., Aono N., Harada M., Okuda T., Iwase A., Kato H., Kudo A. (2017). Development of Various
Metal Sulfide Photocatalysts Consisting of d^0^, d^5^, and d^10^ Metal Ions for Sacrificial H_2_ Evolution
under Visible Light Irradiation. Chem. Lett..

[29] Yamaguchi Y., Kudo A. (2021). Visible Light
Responsive Photocatalysts Developed by Substitution
with Metal Cations Aiming at Artificial Photosynthesis. Front. Energy.

[30] Yokoyama D., Minegishi T., Jimbo K., Hisatomi T., Ma G., Katayama M., Kubota J., Katagiri H., Domen K. (2010). H_2_ Evolution
from Water on Modified Cu_2_ZnSnS_4_ Photoelectrode
under Solar Light. Appl. Phys.
Express.

[31] Kato T., Hakari Y., Ikeda S., Jia Q., Iwase A., Kudo A. (2015). Utilization of Metal Sulfide Material of (CuGa)_1‑X_Zn_2X_S_2_ Solid Solution with Visible Light Response
in Photocatalytic and Photoelectrochemical Solar Water Splitting Systems. J. Phys. Chem. Lett..

[32] Kaga H., Tsutsui Y., Nagane A., Iwase A., Kudo A. (2015). An Effect
of Ag­(I)-Substitution at Cu Sites in CuGaS_2_ on Photocatalytic
and Photoelectrochemical Properties for Solar Hydrogen Evolution. J. Mater. Chem. A.

[33] Yoshino S., Iwase A., Ng Y. H., Amal R., Kudo A. (2020). Z-Schematic
Solar Water Splitting Using Fine Particles of H_2_-Evolving
(CuGa)_0.5_ZnS_2_ Photocatalyst Prepared by a Flux
Method with Chloride Salts. ACS Appl. Energy
Mater..

[34] Kageshima Y., Shiga S., Ode T., Takagi F., Shiiba H., Htay M. T., Hashimoto Y., Teshima K., Domen K., Nishikiori H. (2021). Photocatalytic and Photoelectrochemical Hydrogen Evolution
from Water over Cu_2_Sn_x_Ge_1‑x_S_3_ Particles. J. Am. Chem. Soc..

[35] Minegishi T., Nishimura N., Kubota J., Domen K. (2013). Photoelectrochemical
Properties of LaTiO_2_N Electrodes Prepared by Particle Transfer
for Sunlight-Driven Water Splitting. Chem. Sci..

[36] Ng Y. H., Iwase A., Kudo A., Amal R. (2010). Reducing Graphene Oxide
on a Visible-Light BiVO_4_ Photocatalyst for an Enhanced
Photoelectrochemical Water Splitting. J. Phys.
Chem. Lett..

[37] Ng Y. H., Iwase A., Bell N. J., Kudo A., Amal R. (2011). Semiconductor/Reduced
Graphene Oxide Nanocomposites Derived from Photocatalytic Reactions. Catal. Today.

[38] Iwase A., Ng Y. H., Amal R., Kudo A. (2015). Solar Hydrogen Evolution
Using a CuGaS_2_ Photocathode Improved by Incorporating Reduced
Graphene Oxide. J. Mater. Chem. A.

[39] Takayama, T. ; Iwase, A. ; Kudo, A. Effect of Modification of Polypyrrole on Active Metal Sulfide Photoelectrodes for CO_2_ Reduction under Visible Light Irradiation. In96th CSJ Annual Meeting, 2016.

[40] Takayama T., Iwase A., Kudo A. (2024). Enhancing Photocathodic
Performances
of Particulate-CuGaS_2_-Based Photoelectrodes via Conjugation
with Conductive Organic Polymers for Efficient Solar-Driven Hydrogen
Production and CO_2_ Reduction. ACS
Appl. Mater. Interfaces.

[41] Kameyama T., Osaki T., Okazaki K. I., Shibayama T., Kudo A., Kuwabata S., Torimoto T. (2010). Preparation
and Photoelectrochemical
Properties of Densely Immobilized Cu_2_ZnSnS_4_ Nanoparticle
Films. J. Mater. Chem..

[42] Tsuji I., Shimodaira Y., Kato H., Kobayashi H., Kudo A. (2010). Novel Stannite-Type
Complex Sulfide Photocatalysts A^I^
_2_-Zn-A^IV^-S_4_ (A^I^ = Cu and Ag;
A^IV^ = Sn and Ge) for Hydrogen Evolution under Visible-Light
Irradiation. Chem. Mater..

[43] Łapkowski M., Proń A. (2000). Electrochemical
Oxidation of Poly­(3,4-Ethylenedioxythiophene)
- `in Situ’ Conductivity and Spectroscopic Investigations. Synth. Met..

[44] Nagatsuka K., Yoshino S., Yamaguchi Y., Kudo A. (2025). Facile Z-Scheme Photocatalyst
Sheets for Water Splitting Combined with Visible Light-Responsive
Metal Sulfides and PEDOT of a Conductive Polymer. Energy Fuels.

[45] Dietrich M., Heinze J., Heywang G., Jonas F. (1994). Electrochemical and
Spectroscopic Characterization of Polyalkylenedioxythiophenes. J. Electroanal. Chem..

[46] Zozoulenko I., Singh A., Singh S. K., Gueskine V., Crispin X., Berggren M. (2019). Polarons, Bipolarons,
and Absorption Spectroscopy of
PEDOT. ACS Appl. Polym. Mater..

[47] Yoshino S., Iwase A., Yamaguchi Y., Suzuki T. M., Morikawa T., Kudo A. (2022). Photocatalytic CO_2_ Reduction Using Water as an Electron
Donor under Visible Light Irradiation by Z-Scheme and Photoelectrochemical
Systems over (CuGa)_0.5_ZnS_2_ in the Presence of
Basic Additives. J. Am. Chem. Soc..

[48] Yang W., Moehl T., Service E., Tilley S. D. (2021). Operando Analysis
of Semiconductor Junctions in Multi-Layered Photocathodes for Solar
Water Splitting by Impedance Spectroscopy. Adv.
Energy Mater..

[49] Xia M., Pan L., Liu Y., Gao J., Li J., Mensi M., Sivula K., Zakeeruddin S. M., Ren D., Grätzel M. (2023). Efficient
Cu_2_O Photocathodes for Aqueous Photoelectrochemical CO_2_ Reduction to Formate and Syngas. J.
Am. Chem. Soc..

[50] Levcenko S., Syrbu N. N., Tezlevan V. E., Arushanov E., Doka-Yamigno S., Schedel-Niedrig T., Lux-Steiner M. C. (2007). Optical
Spectra and Energy Band Structure of Single Crystalline CuGaS_2_ and CuInS_2_. J. Phys.-Condes.
Matter.

[51] Nayebi P., Mirabbaszadeh K., Shamshirsaz M. (2013). Density Functional Theory of Structural,
Electronic and Optical Properties of CuXY_2_ (X = In, Ga
and Y = S, Se) Chalcopyrite Semiconductors. Physica B.

