# Supplementary material for: Hybrid PEDOT Conductive Polymer-Powdered Metal Sulfide Photocathodes for Photoelectrochemical Green H2 Production
Source: ACS Appl Mater Interfaces. 2025 Dec 18;18(6):9625–36. doi: 10.1021/acsami.5c15848 (PMC12926948; doi:10.1021/acsami.5c15848)
Supplement: Supplementary file 1 [file am5c15848_si_001.pdf]

Supplementary Information

Hybrid PEDOT Conductive Polymer–Powdered Metal Sulfide Photocathodes for Photoelectrochemical Green H_2 Production

Kengo Nagatsuka[a], Shunya Yoshino[a], Yuichi Yamaguchi[a,b] and Akihiko Kudo[a,b *]

[a]*Department of Applied Chemistry, Faculty of Science, Tokyo University of Science, 1-3 Kagurazaka, Shinjuku-ku, Tokyo 162-8601, Japan*

[b]*Carbon Value Research Center, Research Institute for Science and Technology, Tokyo University of Science, Noda-shi, Chiba-ken 278-8510, Japan*

*Corresponding author: Department of Applied Chemistry, Faculty of Science, Tokyo University of Science, 1-3 Kagurazaka, Shinjuku-ku, Tokyo, 162-8601, Japan.

TEL: +81-3-5228-8267

FAX: +81-3-5261-4631

E-mail: a-kudo@rs.tus.ac.jp

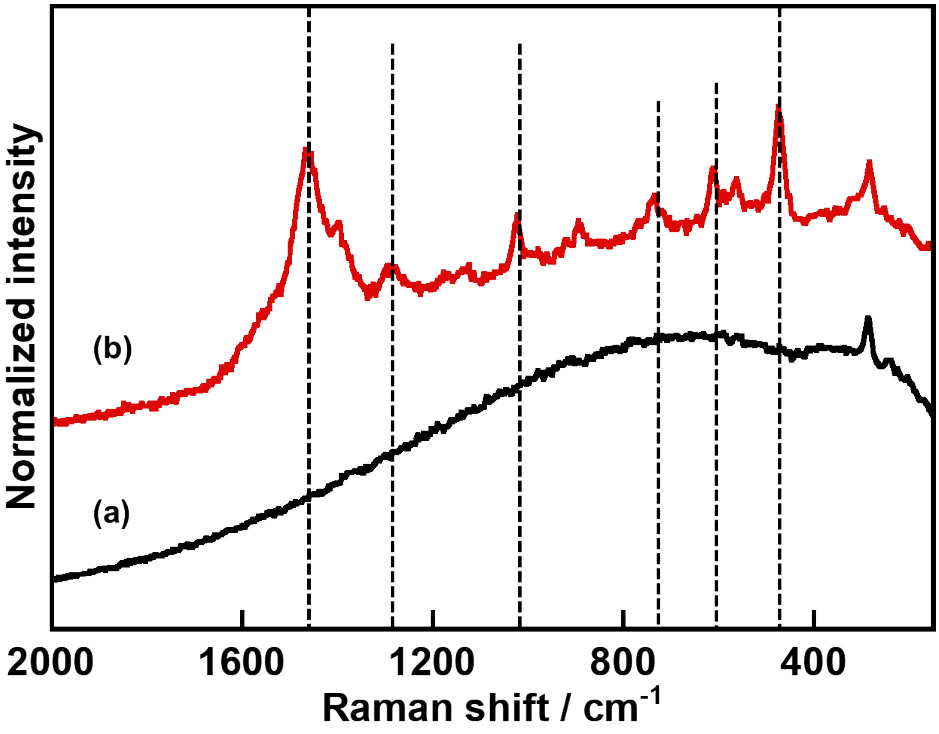

Figure S1. Raman spectra of $(CuGa)_{0.5}ZnS_2$ (flux) photocathodes (a) without and (b) with PEDOT modification. Photocatalyst: 2.0 mg cm^{-2}; PEDOT: 40 mC cm^{-2}; light source: semiconductor laser (λ = 785 nm); dashed line: PEDOT; normalized at 310 cm^{-1} (the Raman peak of $(CuGa)_{0.5}ZnS_2$).

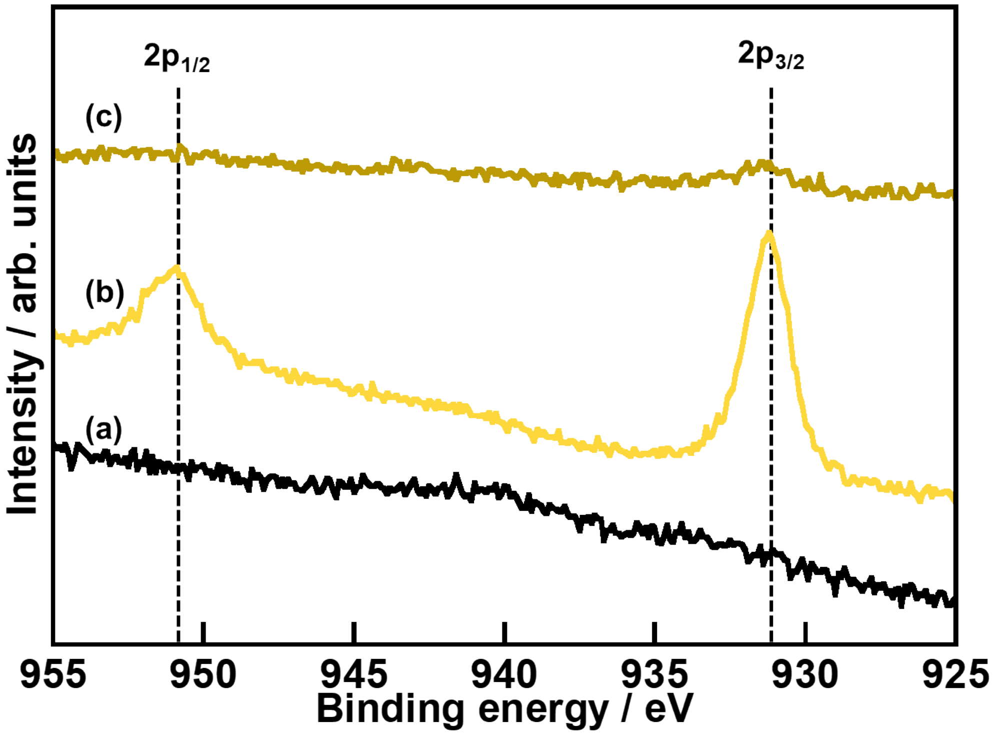

Figure S2. XPS spectra of Cu 2p signals of (a) an FTO substrate and $(CuGa)_{0.5}ZnS_2$ (flux) photocathodes (b) without and (c) with PEDOT modification. Photocatalyst: 2.0 mg cm^{-2}; PEDOT: 40 mC cm^{-2}.

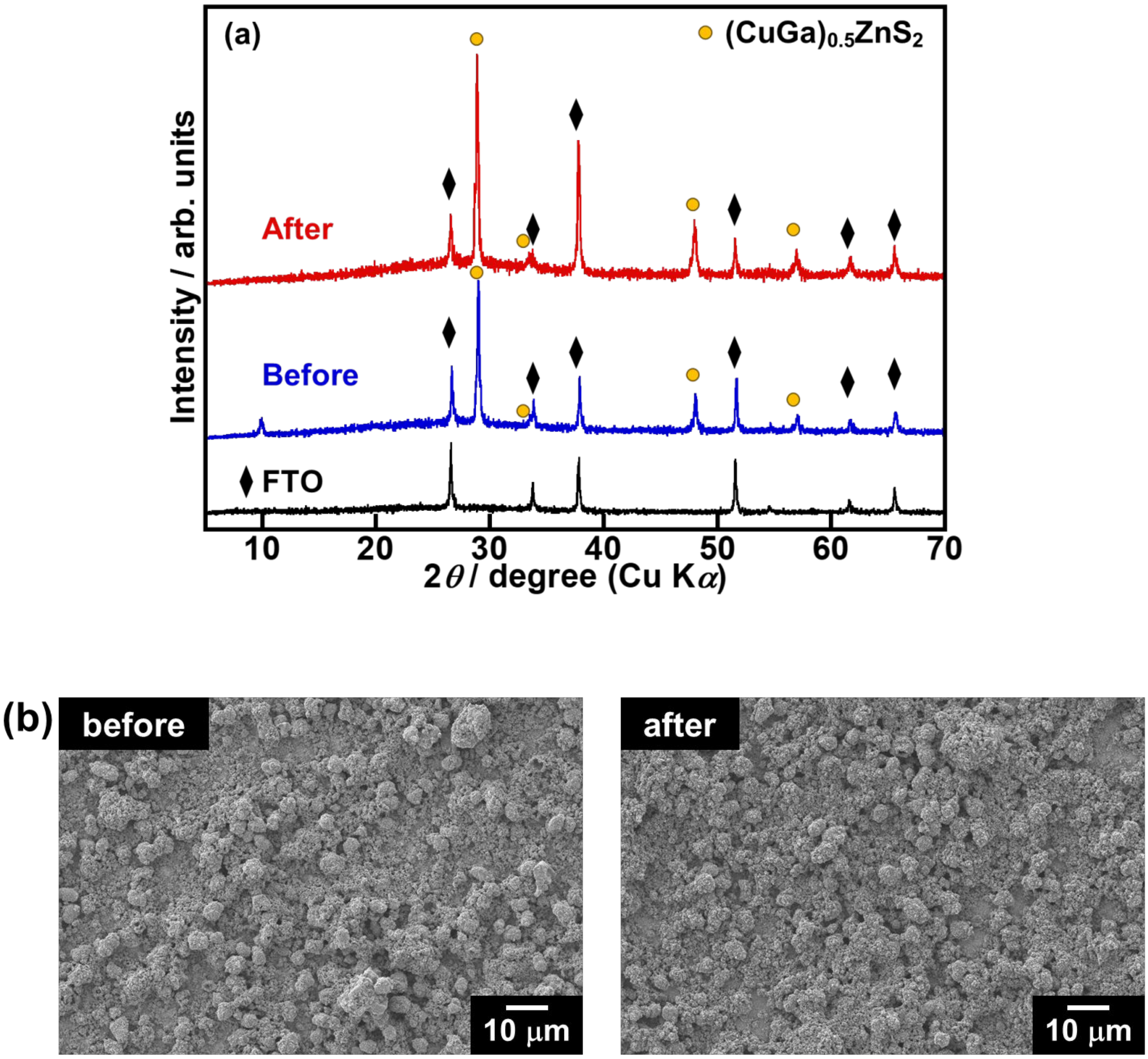

Figure S3. (a) XRD patterns and (b) top-view SEM images of the PEDOT-$(CuGa)_{0.5}ZnS_2$ (flux) photocathode before and after the long-term CA measurement corresponding to Figure 5a.

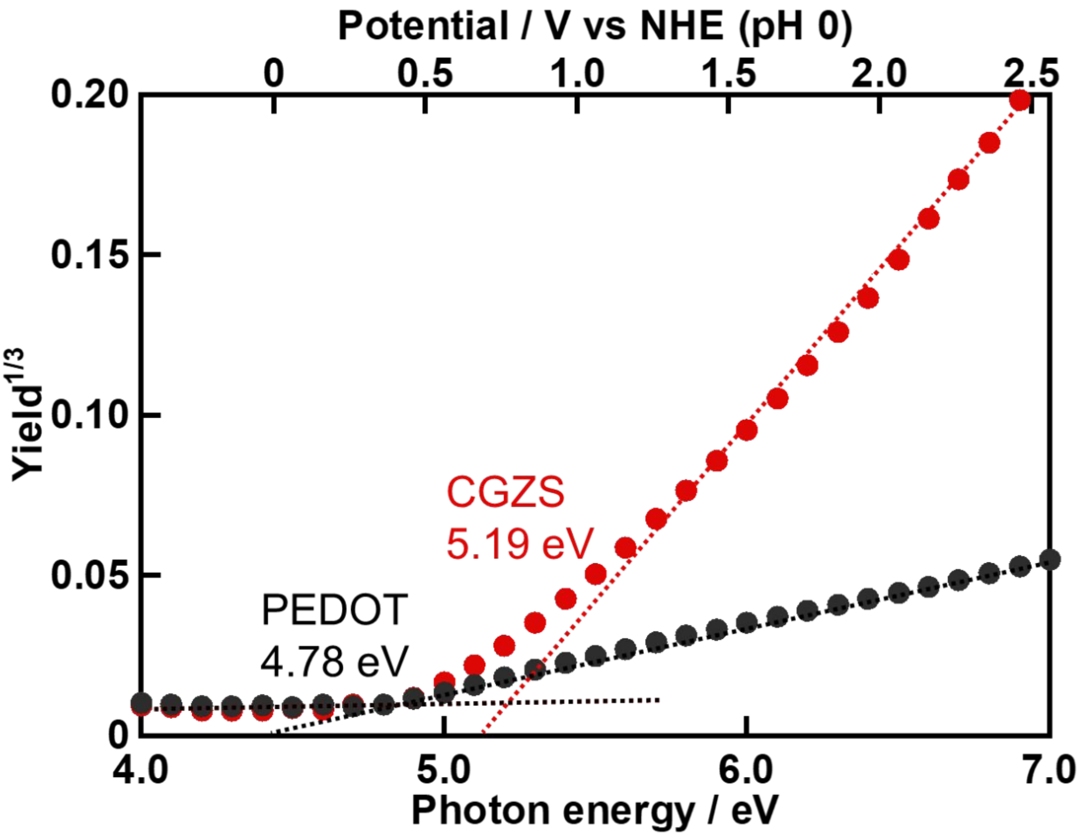

Figure S4. PYS spectra of $(CuGa)_{0.5}ZnS_2$ (flux) (denoted as CGZS) and PEDOT.

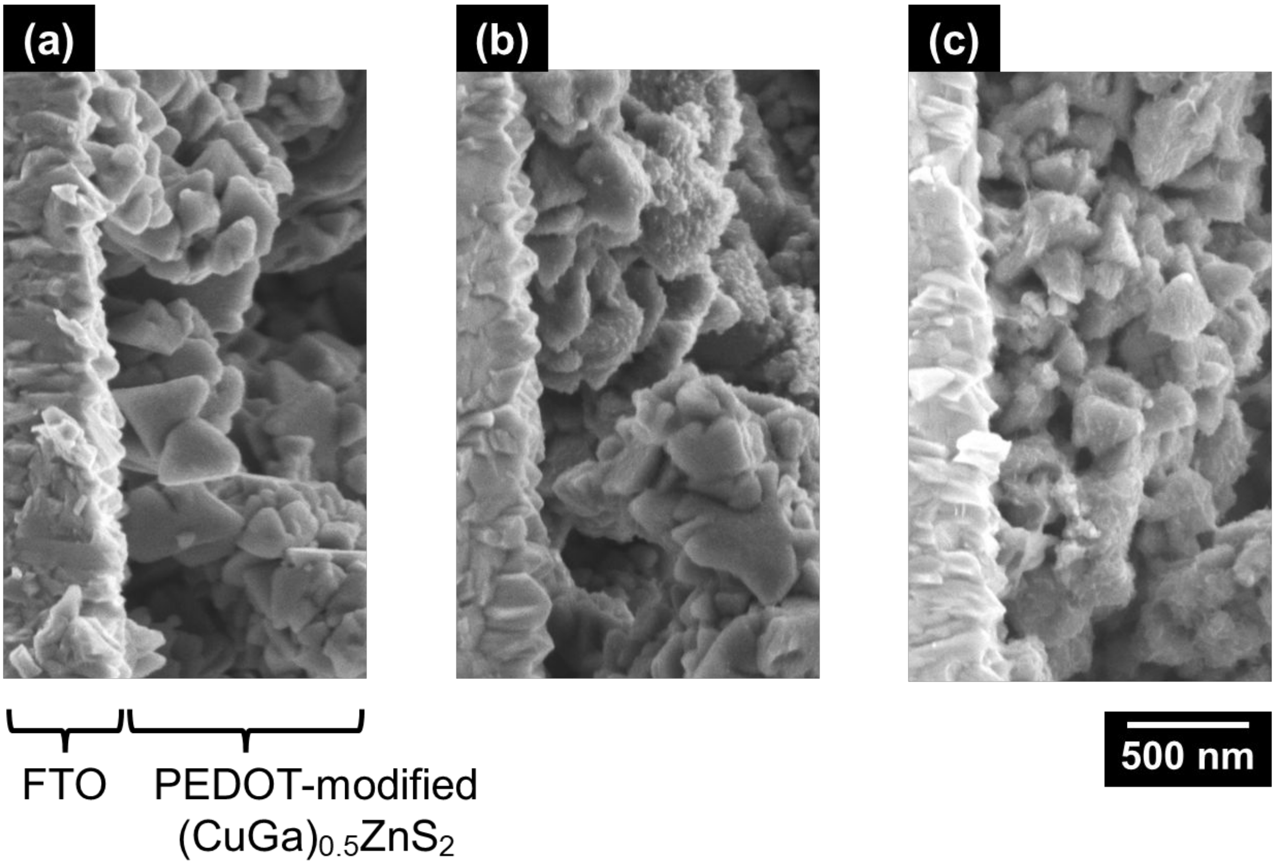

Figure S5. Cross-sectional SEM images of the bottom part of PEDOT-$(CuGa)_{0.5}ZnS_2$ (flux) photocathodes. Photocatalyst: 0.5 mg cm^{-2}; PEDOT: (a) 20, (b) 50, (c) 80 mC cm^{-2}.

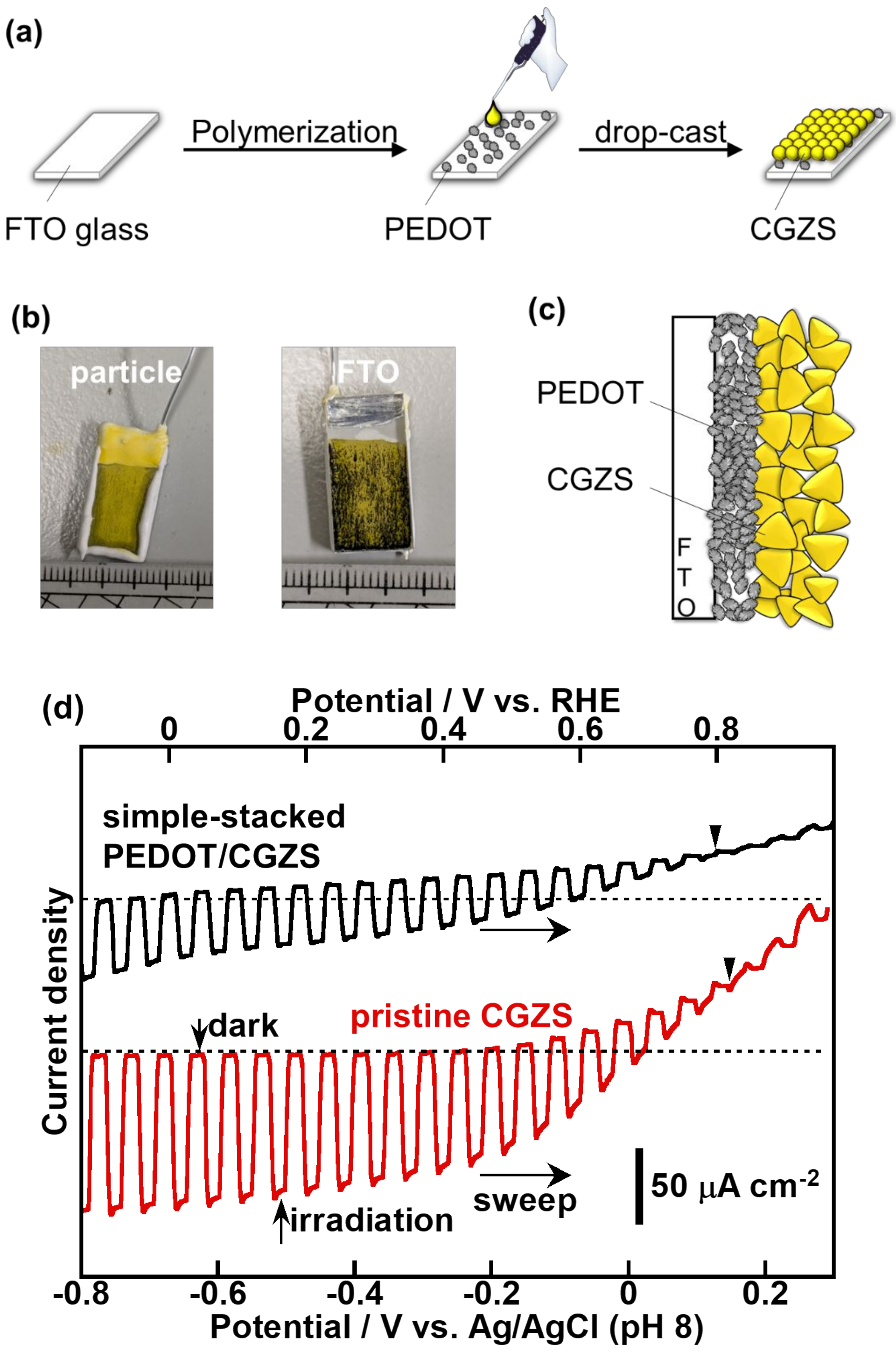

Figure S6. (a) Fabrication scheme, (b) photographs, and (c) schematic illustration of a simple-stacked PEDOT/$(CuGa)_{0.5}ZnS_2$ (flux) photocathode. (d) *J–V* curves of a $(CuGa)_{0.5}ZnS_2$ (flux) photocathode without PEDOT modification and a simple-stacked PEDOT/$(CuGa)_{0.5}ZnS_2$ (flux) photocathode. Photocatalyst: 0.5 mg cm^{-2}; PEDOT: 50 mC cm^{-2}; electrolyte: 0.1 mol L^{-1} $K_2SO_{4\,(aq.)}$ containing a phosphate buffer (pH 8.0) under 1 atm of N_2 gas; CE: Pt wire; RE: Ag/AgCl; scan rate: 20 mV s^{-1}; light source: 300 W Xe lamp (λ > 420 nm), irradiated from an FTO side; cell: H-type cell separated with a Nafion.

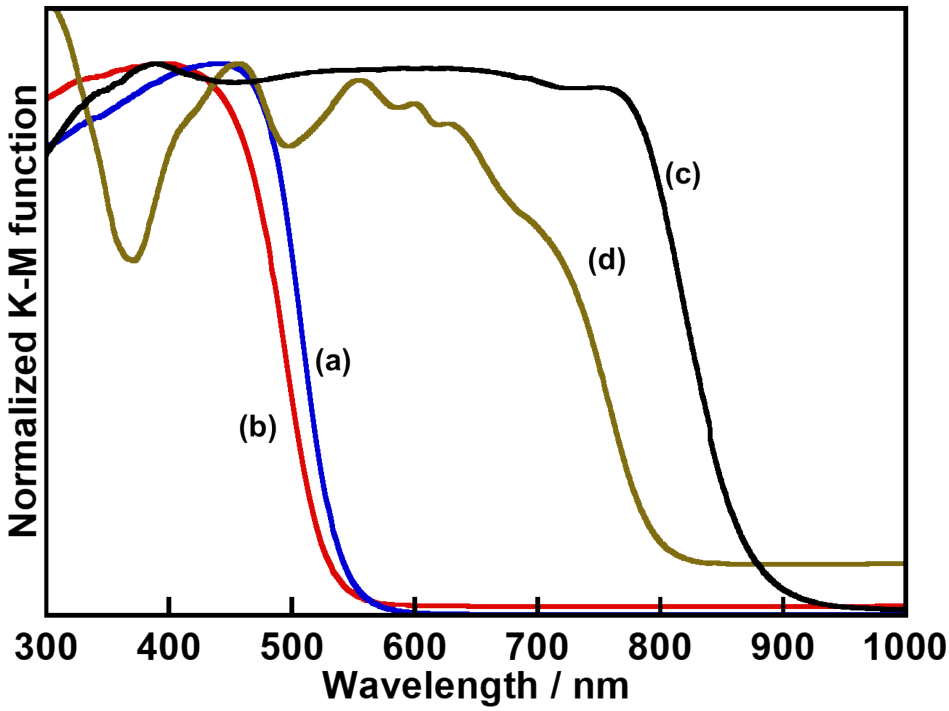

Figure S7. Diffuse reflectance spectra of (a) $(CuGa)_{0.5}ZnS_2$ (SSR), (b) $(CuGa)_{0.5}ZnS_2$ (flux), (c) Cu_2ZnSnS_4 and (d) Cu_3VS_4 powders.

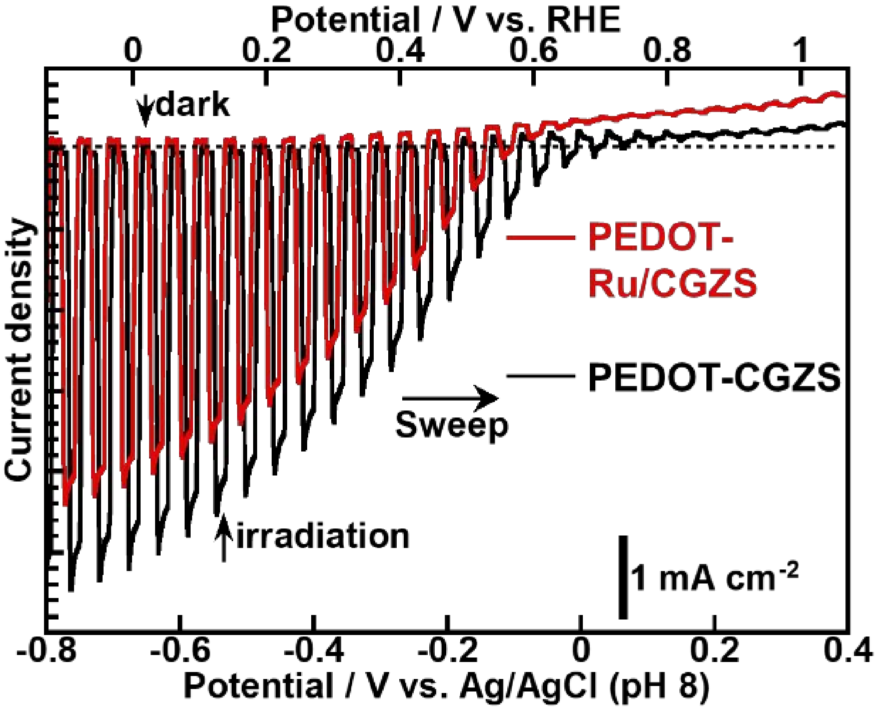

Figure S8. *J–V* curves of PEDOT-$(CuGa)_{0.5}ZnS_2$ (flux) photocathode loaded with a Ru cocatalyst under visible light irradiation. Photocatalyst: 0.5 mg cm^{-2}; cocatalyst: Ru (0.5 wt%, photodeposition); PEDOT: 50 mC cm^{-2}; electrolyte: 0.1 mol L^{-1} $K_2SO_{4\,(aq.)}$ containing a phosphate buffer (pH 8.0) under 1 atm of N_2; CE: Pt wire; RE: Ag/AgCl; scan rate: 20 mV s^{-1}; light source: 300 W Xe lamp ($\lambda > 420$ nm), irradiated from an FTO side; cell: H-type cell separated with a Nafion.

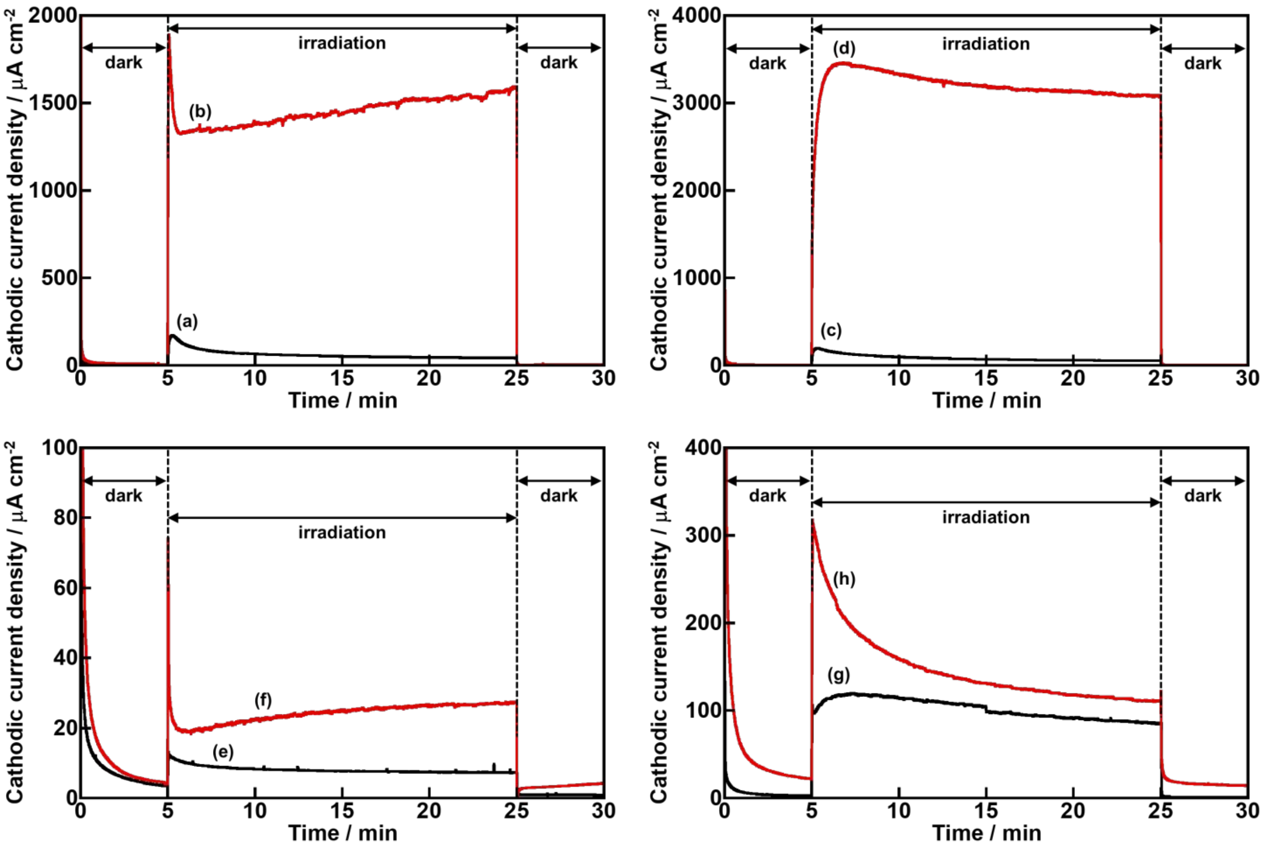

Figure S9. Cathodic current densities under visible light irradiation of metal sulfide photocathodes (a, c, e, g) without and (b, d, f, h) with PEDOT modification. (a, b) $(CuGa)_{0.5}ZnS_2$ (SSR), (c, d) $(CuGa)_{0.5}ZnS_2$ (flux), (e, f) Ru/Cu_2ZnSnS_4 and (g, h) Ru/Cu_3VS_4. Photocatalyst: 2.0 mg cm^{-2}; PEDOT: 40 mC cm^{-2}; cocatalyst: Ru (0.5 wt%, photodeposition); electrolyte: 0.1 mol L^{-1} $K_2SO_{4\,(aq.)}$ containing a phosphate buffer (pH 8.0) under 1 atm of N_2 gas; CE: Pt wire; RE: Ag/AgCl; applied bias: 0 V vs. RHE; light source: 300 W Xe lamp (λ > 420 nm), irradiated from an FTO side; cell: H-type cell separated with a Nafion.

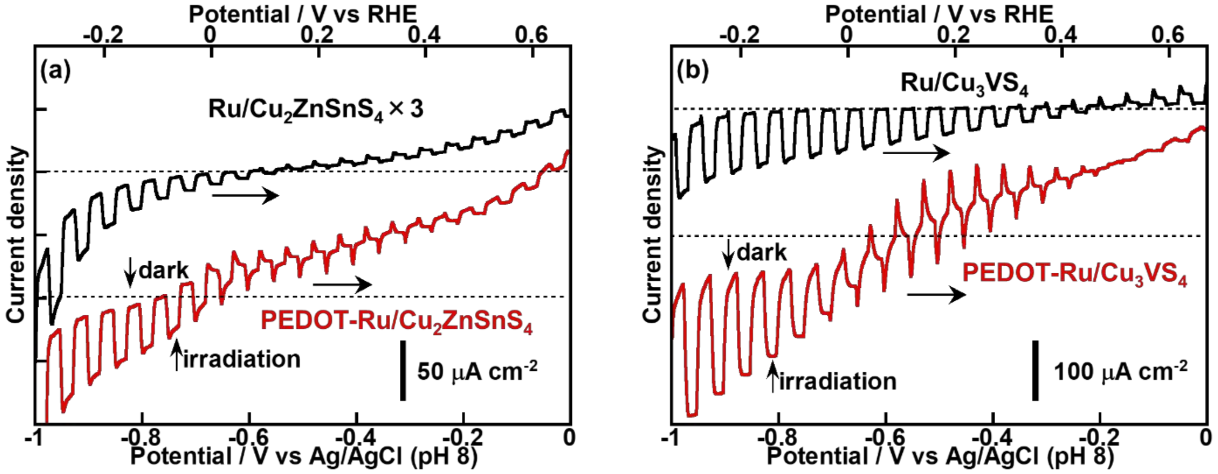

Figure S10. *J*–*V* curves of (a) Ru/Cu_2ZnSnS_4 and (b) Ru/Cu_3VS_4 photocathodes modified with and without PEDOT under visible light irradiation. Photocatalyst: 2.0 mg cm^{-2}; cocatalyst: Ru (0.5 wt%, photodeposition); PEDOT: 40 mC cm^{-2}; electrolyte: 0.1 mol L^{-1} $K_2SO_{4\ (aq.)}$ containing a phosphate buffer (pH 8.0) under 1 atm of N_2; CE: Pt wire; RE: Ag/AgCl; scan rate: 20 mV s^{-1}; light source: 300 W Xe lamp ($\lambda > 420$ nm), irradiated from an FTO side; cell: H-type cell separated with a Nafion.

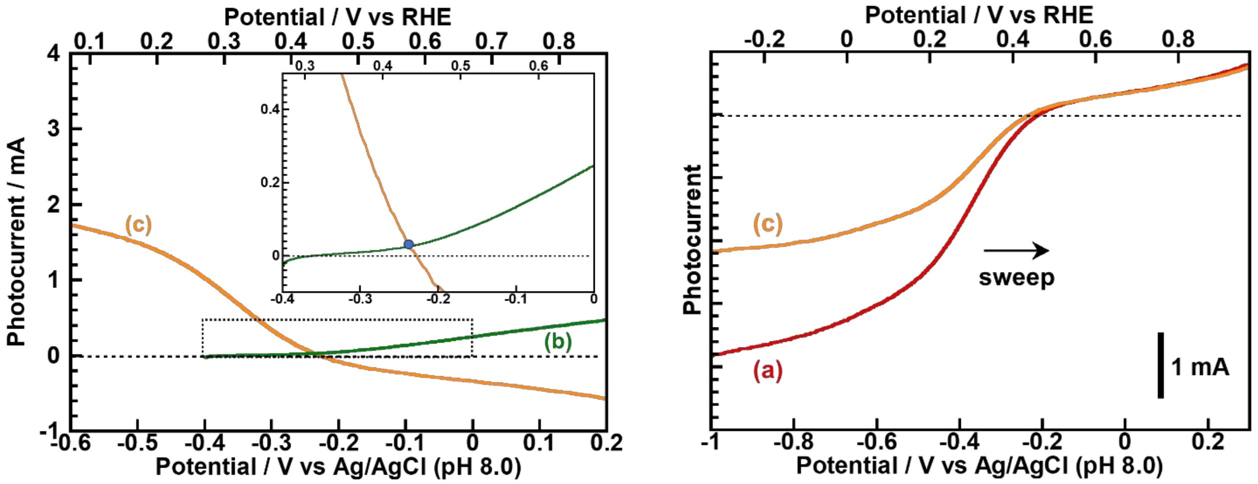

Figure S11. J–V curves of (a) a PEDOT-$(CuGa)_{0.5}ZnS_2$ (flux) photocathode, (b) a $CoO/BiVO_4$:Mo photoanode, and (c) a PEDOT-$(CuGa)_{0.5}ZnS_2$ (flux) photocathode behind a $CoO/BiVO_4$:Mo photoanode under simulated sunlight irradiation. Photocathode: 0.5 mg cm^{-2}; PEDOT: 50 mC cm^{-2}; photoanode: 5.0 µL cm^{-2}; cocatalyst: CoO (8 nmol cm^{-2}, 673 K-1 h in air); electrolyte: 0.1 mol L^{-1} $K_2SO_{4\,(aq.)}$ containing a phosphate buffer (pH 8.0) under 1 atm of Ar gas; CE: Pt wire; RE: Ag/AgCl; scan rate: 20 mV s^{-1}; light source: solar simulator (AM-1.5 G), irradiated from an FTO side; cell: H-type cell separated with a Nafion.

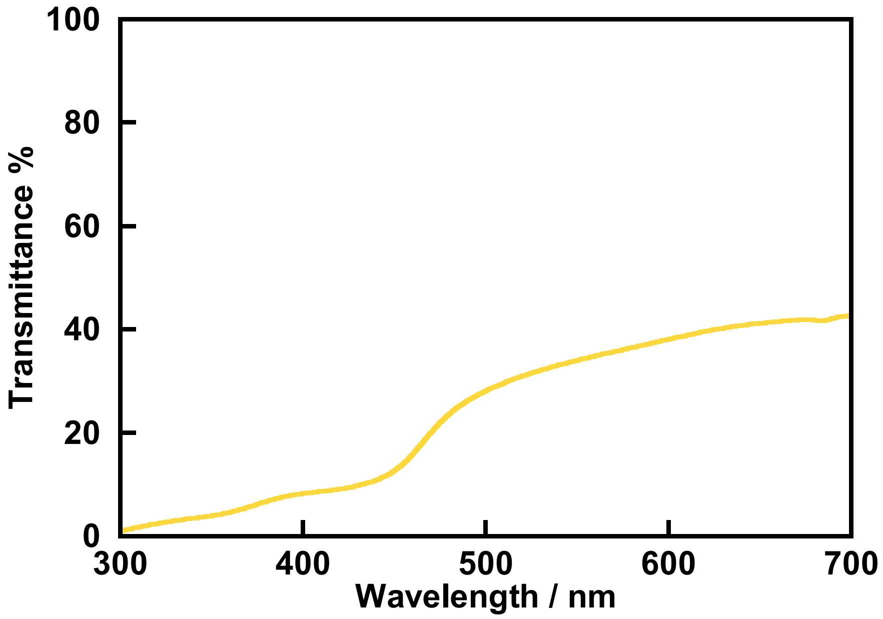

Figure S12. A transmittance spectrum of a CoO/$BiVO_4$:Mo thin-film photoanode. Photoanode: 5.0 μL cm^{-2}; cocatalyst: CoO (8 nmol cm^{-2}, 673 K-1 h in air).
